# The BAF and PRC2 Complex Subunits Dpf2 and Eed Antagonistically Converge on Tbx3 to Control ESC Differentiation

**DOI:** 10.1016/j.stem.2018.12.001

**Published:** 2019-01-03

**Authors:** Wensheng Zhang, Constantinos Chronis, Xi Chen, Heyao Zhang, Rapolas Spalinskas, Mercedes Pardo, Liangliang Chen, Guangming Wu, Zhexin Zhu, Yong Yu, Lu Yu, Jyoti Choudhary, Jennifer Nichols, Mana M. Parast, Boris Greber, Pelin Sahlén, Kathrin Plath

**Affiliations:** 1 Cam-Su Genomic Resource Center, Soochow University, Suzhou 215123, China; 2 Wellcome Sanger Institute, Hinxton CB10 1SA, UK; 3 Department of Biological Chemistry, David Geffen School of Medicine, University of California Los Angeles, Los Angeles, CA, USA; 4 Eli and Edythe Broad Center of Regenerative Medicine and Stem Cell Research, University of California Los Angeles, Los Angeles, CA, USA; 5 Bioinformatics Program, Jonsson Comprehensive Cancer Center, University of California Los Angeles, Los Angeles, CA, USA; 6 Molecular Biology Institute, University of California Los Angeles, Los Angeles, CA 90095, USA; 7 Science for Life Laboratory, Division of Gene Technology, KTH Royal Institute of Technology, 106 91 Stockholm, Sweden; 8 The Institute of Cancer Research, Chester Beatty Laboratories, London, UK; 9 Department of Cell and Developmental Biology, Max Planck Institute for Molecular Biomedicine, Röntgenstrasse 20, 48149 Münster, Germany; 10 Wellcome Trust – Medical Research Council Stem Cell Institute, University of Cambridge, Tennis Court Road, Cambridge CB2 1QR, UK; 11 Department of Pathology, University of California, San Diego, La Jolla, CA, USA; 12 Sanford Consortium for Regenerative Medicine, University of California, San Diego, La Jolla, CA, USA

**Keywords:** pluripotency, self-renewal, cell fate decision, enhancers, differentiation, BAF complex, PRC2 complex, embryonic stem cells, histone modification

## Abstract

BAF complexes are composed of different subunits with varying functional and developmental roles, although many subunits have not been examined in depth. Here we show that the Baf45 subunit Dpf2 maintains pluripotency and ESC differentiation potential. Dpf2 co-occupies enhancers with Oct4, Sox2, p300, and the BAF subunit Brg1, and deleting Dpf2 perturbs ESC self-renewal, induces repression of Tbx3, and impairs mesendodermal differentiation without dramatically altering Brg1 localization. Mesendodermal differentiation can be rescued by restoring Tbx3 expression, whose distal enhancer is positively regulated by Dpf2-dependent H3K27ac maintenance and recruitment of pluripotency TFs and Brg1. In contrast, the PRC2 subunit Eed binds an intragenic Tbx3 enhancer to oppose Dpf2-dependent Tbx3 expression and mesendodermal differentiation. The PRC2 subunit Ezh2 likewise opposes Dpf2-dependent differentiation through a distinct mechanism involving Nanog repression. Together, these findings delineate distinct mechanistic roles for specific BAF and PRC2 subunits during ESC differentiation.

## Highlights

• Dpf2 and Eed antagonistically regulate mesendodermal differentiation of ESCs via Tbx3
• Dpf2 and Eed bind different Tbx3 enhancers to control its expression
• Dpf2 controls H3K27ac and the access of pluripotency TFs at critical target sites
• Ezh2 and Eed counteract Dpf2 function in differentiation through distinct mechanisms

## Graphical Abstract

**Figure:**
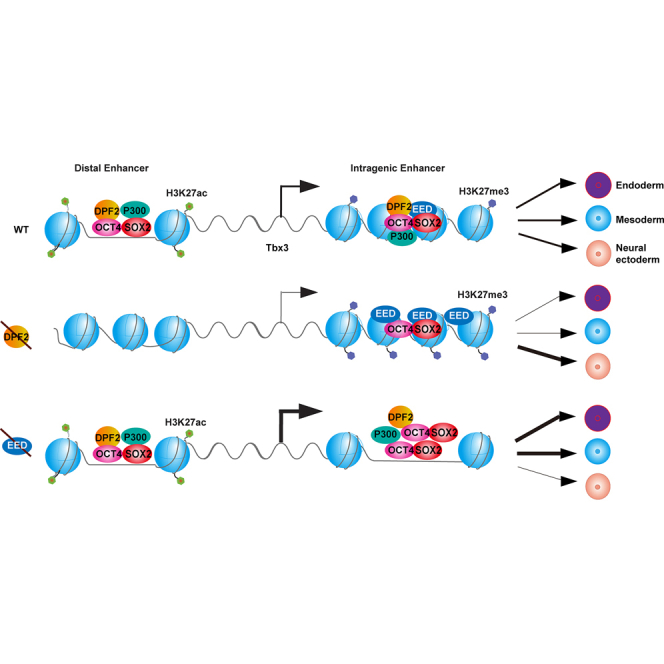

## Teaser

Zhang et al. show that the BAF subunit Dpf2 and the PRC2 subunit Eed antagonistically regulate mesendodermal ESC differentiation by binding distinct Tbx3 enhancers. The PRC2 subunit Ezh2 also counteracts Dpf2 functions in differentiation but through a distinct mechanism involving Nanog repression, revealing that distinct BAF and PRC2 subunits control ESC differentiation.

## Introduction

Embryonic stem cells (ESCs) are capable of self-renewal and differentiation into all cell types of the body, which is conferred by the coordination of key factors, including transcription factors (TFs), polycomb complexes, microRNAs, and histone modifiers (Tee and Reinberg, 2014, Li and Belmonte, 2017). Such factors also include ATP-dependent chromatin remodeling complexes that hydrolyze ATP to change the conformation of chromatin, thereby modulating the access of TFs to chromosomal DNA (Kadoch and Crabtree, 2015). The mammalian switch/sucrose nonfermentable (SWI-SNF) complex, also called the BAF (Brg or Brahma-associated factors) complex, represents one subfamily of the ATP-dependent chromatin remodeling superfamily and forms polymorphic assemblies of up to 15 subunits with different functional specificity based on subunit composition (Kadoch and Crabtree, 2015). BAF complexes have been shown to be essential for mammalian pre- to post-implantation development (Ho and Crabtree, 2010, Panamarova et al., 2016),and play important roles in controlling the self-renewal and pluripotency of ESCs (Ho and Crabtree, 2010). However, the function of only a small number of BAF complex subunits has been studied in ESCs and in the early embryo, and how BAF complexes mechanistically control cell fate decisions is not well understood.

The BAF45 subunit is encoded by a family of four genes (*BAF45a*, *BAF45b*, *BAF45c*, and *BAF45d*) that have different expression patterns (Kadoch and Crabtree, 2015). These proteins contain two plant homeodomain (PHD) fingers that may target the BAF complex to genomic loci bearing specific histone marks (Kadoch and Crabtree, 2015). In the mouse, *BAF45a* is essential for the maintenance of hematopoietic stem cells (Krasteva et al., 2017) and for the self-renewal of neural progenitors and is replaced by *BAF45b*/*c* as neural progenitors differentiate (Kadoch and Crabtree, 2015), whereas *BAF45c* is critical for heart and muscle development (Lange et al., 2008). *BAF45d*, also called *Dpf2*, is the only ubiquitously expressed BAF45 subunit (Mertsalov et al., 2000) and, so far, has been implicated in the programmed cell death response after deprivation of interleukin-3 from myeloid cells (Gabig et al., 1994). However, the biochemical interaction of DPF2 with pluripotency TFs in ESCs (Pardo et al., 2010, van den Berg et al., 2010) suggests a function of this BAF subunit in pluripotent cells, which has not been examined to date.

Our study shows that deletion of *Dpf2* in mouse ESCs decreased their self-renewal ability and dramatically impaired their differentiation into mesoderm and endoderm while promoting neural ectoderm differentiation. The differentiation defect to meso-endoderm could be rescued by restoring *Tbx3* levels in *Dpf2*^*−/−*^ ESCs. We also found that the PRC2 complex subunit *Eed* oppositely regulates meso-endoderm differentiation compared with *Dpf2*, also by regulating *Tbx3* expression. Mechanistically, *Dpf2* and *Eed* act on two different Tbx3-controlling enhancers. We further demonstrate that *Ezh2*, another PRC2 subunit, also regulates meso-endoderm differentiation as opposed to *Dpf2* but through a distinct mechanism that involves *Nanog* suppression. Thus, our work uncovers complex mechanisms by which PRC2 subunits and the BAF subunit *Dpf2* control differentiation of ESCs.

## Results

### *Dpf2* Loss Affects ESC Self-Renewal and Leads to Increased Apoptosis and Cell-Cycle Defects

Given the previously described biochemical interaction of DPF2 with OCT4 in mouse ESCs (Pardo et al., 2010, van den Berg et al., 2010) and the prominent role of OCT4 as a member of the core pluripotency network (Li and Belmonte, 2017), we set out to study the role of *Dpf2* in ESCs. Specifically, we generated a conditional of *Dpf2* allele in ESCs by adding LoxP sites around exon 4 (Figure S1A). 4-Hydroxytamoxifen (4-OHT) treatment of *Dpf2* ^*fl/fl*^ ESCs resulted in an out-of-frame mutation yielding a complete *Dpf2* knockout (KO) at the protein level (Figure S1B).

**Figure 1:**
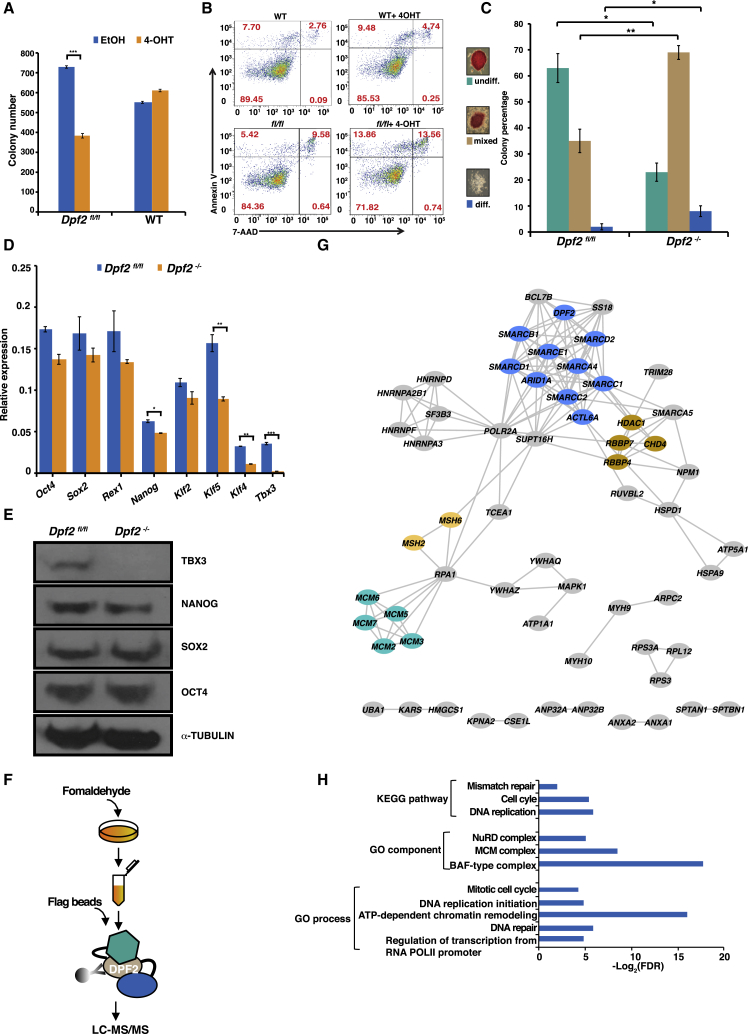
Loss of *Dpf2* Affects ESC Self-Renewal and Leads to Increased Apoptosis and Cell-Cycle Defects (A) Quantification of a colony-formation assay for WT, *Dpf2*^*fl/fl*^, and *Dpf2*^*−/−*^ mouse ESCs. Given is the mean of three replicates and the SD. ^∗∗∗^p < 0.001. (B) Representative fluorescence-activated cell sorting (FACS) plots of Annexin V and 7-aminoactinomycin D (7-AAD) levels in *Dpf2*^*fl/fl*^ and WT control ESCs. Percentages of cells with different apoptosis marker levels are indicated in brackets. (C) Alkaline phosphatase (AP) staining assay for *Dpf2*^*fl/fl*^ and *Dpf2*^*−/−*^ ESCs. Colonies were scored as undifferentiated (undiff), mixed, and differentiated (diff). The mean and SD of three replicates is displayed. ^∗^p < 0.05, ^∗∗^p < 0.01. (D) Transcript levels of pluripotency-associated genes in *Dpf2*^*fl/fl*^ and *Dpf2*^*−/−*^ ESCs based on qPCR. (E) Western blot for OCT4, SOX2, NANOG, and TBX3 protein levels in *Dpf2*^*fl/fl*^ and *Dpf2*^*−/−*^ ESCs; α-TUBB served as a loading control. (F) Schematic of the affinity purification of FLAG-tagged DPF2 in ESCs and the MS procedure. (G) DPF2-interacting proteins annotated in the STRING (Search Tool for the Retrieval of Interacting Genes/Proteins) database. Subunits of the BAF (blue), NuRD (tan), MCM (green), and MSH complexes (yellow) are highlighted. (H) GO term and KEGG (Kyoto Encyclopedia of Genes and Genomes) pathway enrichment of DPF2-interacting proteins. Selected terms are shown. FDR, false discovery rate.

We first tested the role of *Dpf2* in ESC self-renewal. Absence of *Dpf2* expression led to a decrease in colony formation (Figure 1A), suggesting an impairment of self-renewal ability. The lower colony number arising from *Dpf2*^*−/−*^ cells coincided with a small increase in apoptosis under feeder-free conditions in Lif and serum-containing medium because *Dpf2* ^*fl/fl*^ cells were more prone to apoptosis (∼27% cell death) than wild-type (WT) ESCs (∼14%) when treated with 4-OHT for 96 hr (Figure 1B). More significant cell death was also observed for *Dpf2*^*−/−*^ ESCs cultured in N2B27 medium with BMP4 and leukemia inhibitory factor (Lif) (Figure S1C). Additionally, *Dpf2* deletion resulted in an ∼10% increase in cells in the G2-M cell cycle phases, whereas ∼17% fewer cells were present in S phase (Figure S1D). In addition to the decreased ability to form colonies, alkaline phosphatase (AP) staining revealed a decrease in homogeneously stained, undifferentiated colonies in *Dpf2*^*−/−*^ ESCs (Figure 1C). We conclude that increased apoptosis, changes in the cell cycle, and an impaired ability to form colonies are consequences of *Dpf2* deletion in ESCs.

*Dpf2* deletion had no effect on core pluripotency regulators such as *Oct4* and *Sox2* both at the transcript and protein levels (Figures 1D and 1E), indicating that the self-renewal defects observed in *Dpf2*^*−/−*^ ESCs were not associated with precocious differentiation. However, *Nanog* expression decreased slightly at both the transcript and protein levels, and the expression of other pluripotency regulators, including *Tbx3*, *Klf4*, and *Klf5*, was significantly decreased (Figures 1D and 1E). Previous reports have demonstrated the importance of *Tbx3* for the maintenance of ESC self-renewal (Ivanova et al., 2006) and the ability of *Klfs* to support ESC self-renewal (Hall et al., 2009), suggesting that the action of DPF2 on these genes could be critical for ESC self-renewal.

To explore the molecular mechanisms of *Dpf2* in ESCs, we fused an affinity purification (FTAP) tag (Bode et al., 2016) to the C terminus of one endogenous *Dpf2* allele in ESCs (Figures S1E and S1F) and, after cross-linking of cells with formaldehyde to stabilize transient interactions, performed affinity purification of DPF2-containing protein complexes, followed by mass spectrometry (Figure 1F), and confirmed the results with immunoprecipitation followed by western blotting. We identified 80 high-confidence DPF2 interaction partners (Table S1; Figure 1G), including several components of the BAF complex (BRG1 [SMARCA4], ARID1A, BAF155 [SMARCC1], BAF57 [SMARCE1], and BAF170 [SMARCC2]) (Figure S1G), corroborating that DPF2 is a subunit of the BAF complex in ESCs. Gene ontology (GO) analysis of the DPF2 interactome revealed a significant association with proteins that exhibit chromatin-regulatory functions, including the nucleosome remodeling deacetylase (NuRD) complex (Figure 1H). In agreement with the cell cycle defect in *Dpf2*^*−/−*^ ESCs, DPF2 associated with proteins implicated in regulation of the cell cycle and DNA replication initiation (Figures 1G and 1H), including most subunits of the mini chromosome maintenance (MCM) complex, which controls DNA replication (Forsburg, 2004), suggesting that *Dpf2* (and the BAF complex) may affect the ESC cell cycle by regulating the interaction of the MCM complex with DNA replication origins.

### *Dpf2* Deletion Alters ESC Differentiation

**Figure 2:**
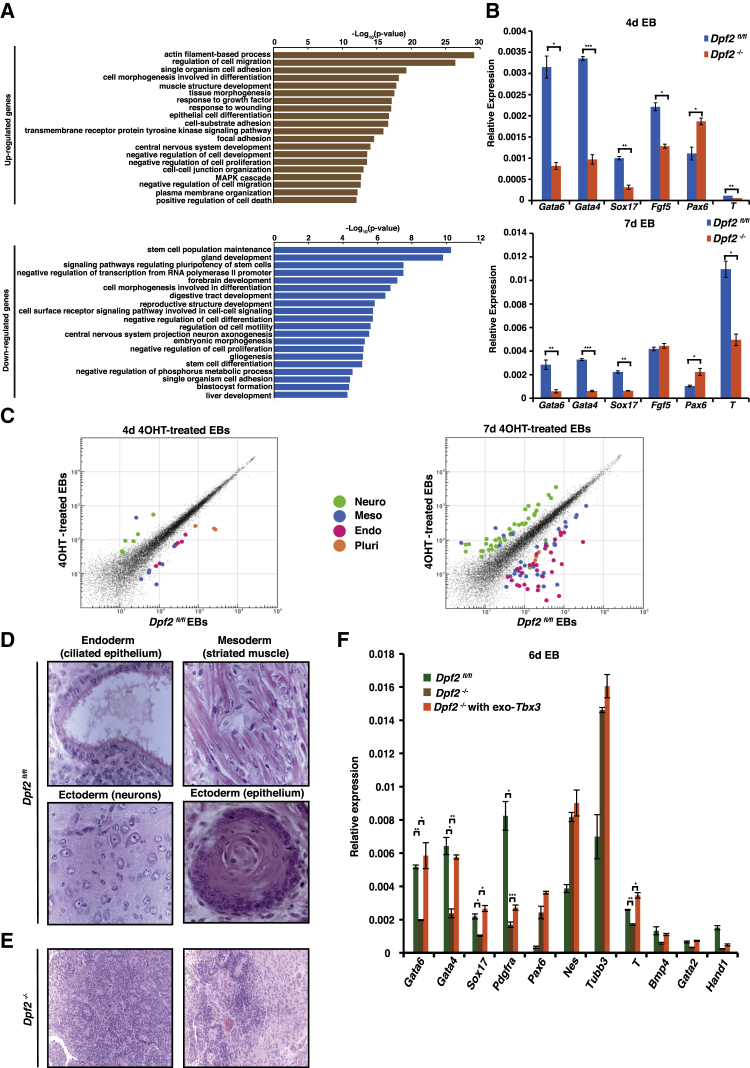
*Dpf2* Deletion Impairs ESC Differentiation (A) GO analysis for biological processes associated with genes differentially expressed upon *Dpf2* deletion in ESCs. (B) qPCR analysis for transcript levels of the indicated lineage-specific genes on days 4 and 7 of EB differentiation of *Dpf2*^*fl/fl*^ and *Dpf2*^*−/−*^ cells. (C) Global gene expression profiles of *Dpf2*^*fl/fl*^ and *Dpf2*^*−/−*^ EBs. Neuro, neural ectoderm markers; Meso, mesoderm markers; Endo, endoderm markers; Pluri, pluripotency-associated genes. (D) Images of H&E-stained teratomas from *Dpf2*^*fl/fl*^ ESCs. Magnification, 630×. (E) As in (D), except for *Dpf2*^*−/−*^ ESCs. Magnification, 200×. (F) qPCR analysis for transcript levels of the indicated lineage-specific genes in day 6 EBs from *Dpf2*^*fl/fl*^, *Dpf2*^*−/−*^, and *Dpf2*^*−/−*^ with ectopically (exo) expressed *Tbx3*.

Because KO of *Dpf2* altered the expression of some pluripotency regulators, we examined the global gene expression changes upon *Dpf2* deletion in ESCs by RNA sequencing (RNA-seq). We identified 383 significantly down- and 753 upregulated genes when comparing *Dpf2*^*fl/fl*^ and *Dpf2*^*−/−*^ ESCs (Figure S2A; Table S2). *Dpf2* loss led to the downregulation of genes associated with stem cell maintenance, blastocyst formation, and signaling pathways that control pluripotency and, in agreement with an increase in apoptosis, induced the upregulation of genes associated with cell death and negative regulation of proliferation (Figure 2A). Interestingly, we identified a number of differentiation-associated GO terms for both down- and upregulated genes in *Dpf2* KO ESCs (Figure 2A), suggesting a role of *Dpf2* in controlling differentiation pathways.

To examine the differentiation potential of *Dpf2*^*−/−*^ ESCs, we performed embryoid body (EB) differentiation assays and assessed the expression level of well-established lineage markers. The transcript levels of the endoderm markers *Gata4*, *Gata6*, and *Sox17* and the mesoderm marker *Brachury* (*T*) were significantly lower in *Dpf2*^*−/−*^ EBs cultured for 4 or 7 days compared with their respective controls (Figure 2B), suggesting that deletion of *Dpf2* leads to impaired endoderm and mesoderm differentiation. The expression of the ectoderm marker *Fgf5* was lower in *Dpf2*^*−/−*^ EBs on day 4 but similar in WT and *Dpf2*^*−/−*^ EBs cultured for 7 days (Figure 2B). Conversely, the expression of the neural ectoderm marker *Pax6* was upregulated throughout EB differentiation, indicating that *Dpf2* loss promoted neural ectoderm differentiation (Figure 2B). We also induced ESC differentiation with retinoic acid (RA), which promotes differentiation into primitive endoderm (Cho et al., 2012). Immunostaining against SOX17, GATA6, and GATA4 in *Dpf2*^*−/−*^ RA-differentiated cells revealed the reduction of these markers compared with WT cells, indicating that differentiation to primitive endoderm is significantly impaired without *Dpf2* (Figure S2B).

To gain more insights into the differentiation bias of *Dpf2*^*−/−*^ ESCs, we examined the global gene expression profile of *Dpf2*^−/−^ EBs and corresponding WT controls. On day 4, a few marker genes for endoderm and mesoderm showed decreased expression and some neural-related genes showed increased expression in mutant EBs compared with controls (Figure 2C, left; Table S3). These differences were even more pronounced in EBs on day 7, when endoderm and mesoderm-related genes showed significantly decreased and neuron-related genes increased expression in *Dpf2*^*−/−*^ EBs (Figure 2C, right; Table S3).

To investigate the effects of *Dpf2* deletion on differentiation *in vivo*, we assayed *Dpf2*^*−/−*^ and *Dpf2*^*fl/fl*^ ESCs for their ability to form teratomas. *Dpf2*^*fl/fl*^ ESCs formed well-differentiated teratomas containing cells of all three germ layers (Figure 2D). Conversely, *Dpf2*^*−/−*^ ESCs formed mostly immature teratomas containing mostly immature neural ectoderm tissue (with prominent neuroepithelial rosettes) and trophoblast giant cells with minimal endoderm- and mesoderm-related tissues (Figures 2E and S2C). Thus, deletion of *Dpf2* alters the differentiation propensity of ESCs *in vitro* and *in vivo*, away from meso-endoderm toward immature neural ectoderm.

To validate that the impaired differentiation phenotype was caused by deletion of the *Dpf2* gene, we asked whether overexpression of FLAG-tagged *Dpf2* rescued the defects (Figure S2D). *Dpf2* overexpression in *Dpf2*^*fl/fl*^ ESCs before disruption of the endogenous *Dpf2* alleles did not affect self-renewal or differentiation (Figures S2E and S2F). However, when endogenous *Dpf2* was deleted in *Dpf2* ^*fl/fl*^ ESCs by 4-OHT, *Dpf2* overexpression rescued the differentiation defects of all three lineages, restoring the levels of lineage-specific markers to WT levels (Figure S2G).

### Specificity of *Dpf2* Function in ESC Differentiation

In addition to *Dpf2* (*BAF45d*), *BAF45a* is expressed in mouse ESCs, whereas *BAF45b* and *BAF45c* are lowly expressed (Kadoch and Crabtree, 2015). To examine whether the differentiation defect observed upon the *Dpf2* deletion is specific for *Dpf2*, we generated *Dpf2*^*fl/fl*^ ESC lines overexpressing *BAF45a* and *BAF45c* (Figure S2H). Overexpression of either subunit prior to the deletion of *Dpf2* did not alter the expression of pluripotency and differentiation marker genes (Figures S2I–S2L). *BAF45a* or *BAF45c* overexpression did not rescue the differentiation defects toward endoderm, mesoderm, and neural ectoderm of cells lacking endogenous *Dpf2* (Figure S2M). These results indicate that the BAF45 subunits are not functionally redundant and highlight a specific role of *Dpf2* in lineage specification from ESCs.

### *Tbx3* Rescues the Meso-endoderm Differentiation Defects of *Dpf2*^−/−^ ESCs

*Tbx3* was one of the pluripotency genes most affected by deletion of *Dpf2* in undifferentiated ESCs (Figure 1E). Considering the requirement of *Tbx3* for meso-endoderm differentiation (Weidgang et al., 2013, Waghray et al., 2015), we hypothesized that *Dpf2* may control the differentiation potential of ESCs via regulation of *Tbx3* expression. To test this idea, we stably transfected FLAG-tagged *Tbx3* into *Dpf2*^*fl/fl*^ cells and subsequently deleted *Dpf2*. The data show that the endoderm marker genes *Gata4*, *Gata6*, *Sox17*, and *Pdgfra* reached nearly WT levels in *Dpf2*^−/−^ 4-day and 6-day EBs ectopically expressing *Tbx3* (Figures 2F and S2N). Similarly, ectopic *Tbx3* expression restored the expression levels of the mesoderm marker genes *T*, *Bmp4*, *Gata2*, and *Hand1* in *Dpf2*^*−/−*^ 6-day EBs (Figures 2F and S2N). Conversely, the increase in neural ectoderm markers (*Pax6*, *Nes*, and *Tubb3*) observed in *Dpf2*^−/−^ EBs was not reduced by Tbx3 overexpression (Figures 2F and S2N). We conclude that overexpression of *Tbx3* rescued the endoderm and mesoderm differentiation defects induced by loss of *Dpf2* and that the enhancement of neural-ectoderm differentiation in *Dpf2*^*−/−*^ EBs did not occur through regulation of *Tbx3*.

Because *Tbx3* overexpression itself promotes endodermal and mesodermal genes in differentiating ESCs (Weidgang et al., 2013), it remained possible that *Tbx3* expression may upregulate endo- and mesodermal genes in cells that have differentiated toward neuroectoderm upon Dpf2 deletion. To exclude this possibility, we performed immunofluorescence staining of GATA4 in combination with NESTIN and TUBB3 in differentiating *Dpf2*^*−/−*^ cells expressing *Tbx3* exogenously and found that cells expressed GATA4 in the absence of the neuroectoderm markers (Figures S2O and S2P). Thus, in the absence of *Dpf2*, *Tbx3* overexpression induces faithful differentiation toward endodermal and mesodermal lineages.

### DPF2 Co-occupies Active Enhancers with OCT4, SOX2, and BRG1 in ESCs

**Figure 3:**
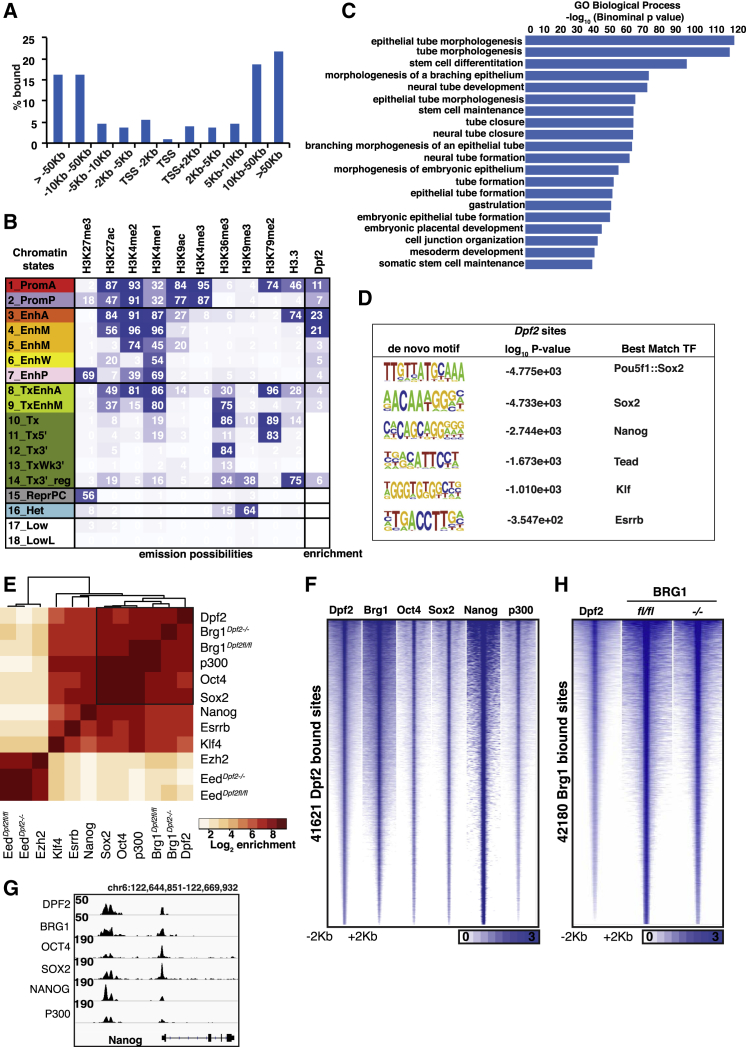
DPF2 Associates with Active Enhancers and the Oct4, Sox2, and Brg1 Network in ESCs (A) Distribution of DPF2 target sites determined by ChIP-seq in ESCs in relation to their distance to TSSs. (B) Chromatin state enrichment of DPF2 target sites in ESCs. ESC chromatin states were defined in Chronis et al. (2017) using ChromHMM. Rows represent chromatin states and their mnemonics. Columns give the frequency of the indicated histone marks and H3.3 for each chromatin state (ChromHMM emission probabilities), color-coded from blue (highest) to white (lowest). Enrichment of DPF2 in each chromatin state is shown in the last column. (C) Significant GO terms for genes with DPF2 target sites within 10 kb of their TSS. (D) Motifs identified at DPF2-bound sites by *de novo* search and the best matching TFs. (E) Hierarchical clustering of pairwise enrichments of DPF2, BRG1, OCT4, SOX2, NANOG, ESRRB, KLF4, EED, and EZH2 binding sites in ESCs. BRG1 and EED binding sites were identified from *Dpf2*^*fl/fl*^ and *Dpf2*^*−/−*^ ESC lines, and all other binding events were obtained from WT ESCs. The black box indicates highest correlation enrichment for pairwise binding. (F) Heatmaps of normalized ChIP-seq signal for Dpf2, Brg1, Oct4, Sox2, Nanog, and p300 at all sites bound by DPF2 in ESCs. (G) Genome browser view of DPF2, BRG1, OCT4, SOX2, NANOG, and P300 binding at the *Nanog* locus in ESCs. (H) Heatmaps of normalized ChIP-seq signal for Dpf2 and Brg1 at BRG1 occupied sites in *Dpf2*^*fl/fl*^ or *Dpf2*^−/−^ ESC lines.

To understand how *Dpf2* regulates *Tbx3*, we profiled the genome-wide binding sites of DPF2 in ESCs using chromatin immunoprecipitation followed by high-throughput sequencing (ChIP-seq) (Table S4). We found that the majority of DPF2 binding events occurred at genomic locations distal to transcriptional start sites (TSSs), including both intergenic regions and gene bodies (Figure 3A). By intersecting DPF2-bound genomic locations with previously annotated ESC chromatin states (Chronis et al., 2017), we found that DPF2 predominantly binds enhancers, particularly those with high levels of the histone marks H3K27ac, H3K4me2, and H3K4me1, characteristic of the most active enhancers (Figure 3B, states 3 and 4). GO analysis indicated that DPF2-occupied sites are located close to genes associated with functions in stem cell maintenance, morphogenesis, gastrulation, and development (Figure 3C).

To gain insights into the determinants of DPF2 binding, we searched for sequence elements enriched within DPF2 target sites. We found motifs for the Klf and Tead family members Esrrb, Nanog, and Sox2 as well as the Oct-Sox composite site to be most enriched (Figure 3D), suggesting that DPF2 is bound at sites engaged by the core pluripotency TFs. This result was corroborated by comparing the binding profile of DPF2 with those of Oct4, Sox2, Nanog, Klf4, and Esrrb genome-wide (Figures 3E and 3F) and at the *Nanog* locus as an example (Figure 3G). Among these pluripotency TFs, DPF2 binding sites overlapped more often with those of Oct4 and Sox2 than with those of Nanog, Esrrb, and Klf4 (Figure 3E). In agreement with previous data showing that DPF2 is a component of the OCT4 protein network (van den Berg et al., 2010, Pardo et al., 2010), we confirmed the interaction of the two proteins by co-immunoprecipitation (Figure S3A) and found that 48.5% of all OCT4 binding events were co-occupied by DPF2.

Additionally, we performed ChIP-seq for BRG1, the ATPase subunit of the BAF complex, and found a high overlap of DPF2 and BRG1 genome occupancy, consistent with mass spectrometry (MS) (Figure 1G) and immunoprecipitation data for DPF2 (Figure S3B) and the notion that DPF2 mainly acts as a subunit of the BAF complex in ESCs (Figures 3E-H). As expected from the localization of DPF2 to active enhancers (Figure 3B), we also detected an extensive co-localization of DPF2 with the transcriptional co-activator P300 (Figures 3E-3G). The significant co-binding between DPF2 and P300 was further supported by the physical interaction observed between DPF2 and P300 in co-immunoprecipitation experiments (Figure S3C). DPF2 binding did not extensively overlap with that of EED and EZH2, subunits of the repressive polycomb complex PRC2 (Figures 3E, S3D, and S3E). Together, these findings reveal collaboration with pluripotency TFs at enhancers and suggest that *Dpf2* plays a role in the selection and activation of enhancers in ESCs.

### *Dpf2* Loss Does Not Globally Affect the Binding of BRG1 and PRC2

To determine whether *Dpf2* deletion globally affects the binding of BRG1, we performed ChIP-seq for BRG1 in ESCs after *Dpf2* deletion. *Dpf2* loss did not prevent binding of BRG1, which maintained a similar genome-wide binding profile as in WT *Dpf2*^*fl/fl*^ ESCs (Figures 3E, 3H, and S3D). This result suggests that the BAF complex assembly remains unperturbed at the vast majority of its physiological targets in the absence of *Dpf2*. Similarly, *Dpf2* loss did not affect the binding profile of the PRC2 subunit EED at the genome-wide level (Figure S3E), consistent with their largely non-overlapping binding sites (Figure 3E).

### *Dpf2* Depletion Modulates H3K27ac Levels and Binding of OCT4 and BRG1

**Figure 4:**
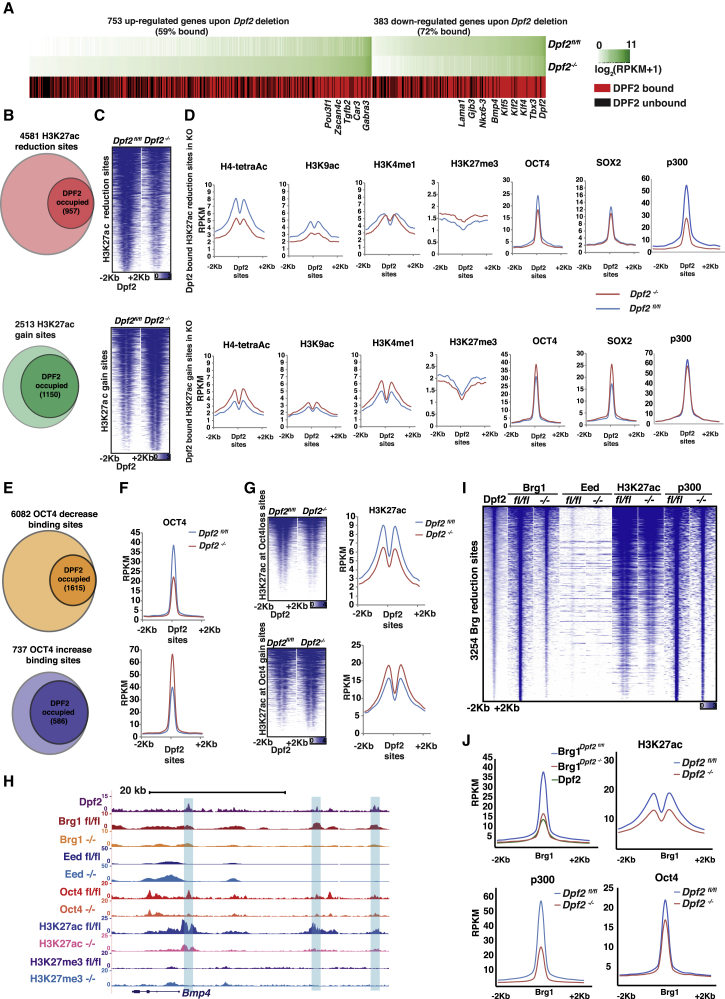
*Dpf2* Depletion Modulates Levels of H3K27ac and OCT4 (A) Heatmap of expression levels (log_2_RPKM+1) in *Dpf2*^*fl/fl*^ and *Dpf2*^*−/−*^ ESCs for differentially expressed genes between *Dpf2*^*fl/fl*^ and *Dpf2*^*−/−*^ ESCs and their DPF2 binding based on a DPF2 peak within 10 kb of the TSS (red, bound; black, unbound genes). (B) Number of genomic sites with a significant change in H3K27ac (>2-fold difference) between *Dpf2*^*fl/fl*^ and *Dpf2*^*−/−*^ ESCs, divided into those with a reduction (top) and increase (bottom) in *Dpf2*^*−/−*^ ESCs, and their association with DPF2 in WT ESCs. (C) Heatmap of normalized tag density profiles of H3K27ac experiments in *Dpf2*^*fl/fl*^*and Dpf2*^*−/−*^ cells at sites with significant H3K27ac changes from (B). (D) Metaplots of average signal intensities for H4tetrac, H3K9ac, H3K4me1, H3K27me3, OCT4, SOX2, and P300 at DPF2-bound sites in ESCs with reduced (top) or increased (bottom) H3K27ac, as defined in (B), for *Dpf2*^*fl/fl*^ (blue) and *Dpf2*^*−/−*^ ESCs (red). (E) As in (B), but for sites with significant differences in OCT4 instead of H3K27ac. (F) Metaplots of average signal intensities for OCT4 in *Dpf2*^*fl/fl*^ and *Dpf2*^*−/−*^ ESCs at sites with significant OCT4 changes that are also occupied by DPF as defined in (E). (G) Heatmap of normalized tag density profiles of H3K27ac e in *Dpf2*^*fl/fl*^ and *Dpf2*^*−/−*^ ESCs at sites with significant OCT4 binding changes and occupied by DPF2 (E) and corresponding metaplots of signal intensities. (H) Genome browser view of ChIP-seq tracks of DPF2, BRG1, EED, and OCT4 binding as well as H3K27ac and H3K27me3 for the *Bmp4* locus. DPF2 data are from WT ESCs and the others from *Dpf2*^*fl/fl*^ and *Dpf2*^*−/−*^ ESCs, indicated as fl/fl and −/−. Regions highlighted in blue signify ESC enhancer regions as defined by ChromHMM in Chronis et al. (2017). The values on the y axis represent fold enrichment over control. (I) Heatmap of normalized tag density profiles of DPF2, EED, H3K27ac, and P300, at sites exhibiting a reduction in BRG1 in *Dpf2*^*−/−*^ ESCs compared with *Dpf2*^*fl/fl*^ ESCs. DPF2 data are from WT ESCs and all others from *Dpf2*^*fl/fl*^ and *Dpf2*^*−/−*^ ESCs, indicated as fl/fl and −/−. (J) Metaplots of average signal intensities for DPF2 and BRG1, H3K27ac, p300 and Oct4 at sites defined in (I).

Given the gene expression changes observed by the absence of *Dpf2*, we investigated whether DPF2 engages genes that become deregulated in KO ESCs. We found that ∼63% of the differentially expressed genes in *Dpf2*^*−/−*^ ESCs associated with a DPF2 binding event within 20 kb around their TSSs (Figure 4A; 719 of 1,136 genes, p < 0.0001, chi-square test), including both up- and downregulated genes, suggesting that *Dpf2* can activate and repress gene expression. Pluripotency genes downregulated in the absence of *Dpf2*, including *Klf4*, *Klf5*, and *Tbx3,* were direct binding targets of DPF2 (Figure 4A).

We next asked how *Dpf2* affects the histone modification landscape. Hence, we generated maps for five histone modifications from *Dpf2*^*fl/fl*^ or *Dpf2*^*−/−*^ ESC lines, including H3K27ac, H3K4me1, H4-tetraAC and H3K9ac, which are associated with promoters and enhancers, and the repressive histone mark H3K27me3 (Ernst et al., 2011). We did not observe significant differences in the average H3K27ac signal between *Dpf2*^*fl/fl*^ and *Dpf2*^*−/−*^ cells when all DPF2 binding sites were considered (Figure S4A). However, we identified 4,581 sites with significant reduction (≥2-fold) of H3K27ac in *Dpf2*^*−/−*^ compared with *Dpf2*^*fl/fl*^ ESCs (Figures 4B and 4C). We also observed 2,513 sites that gained H3K27ac signal by 2-fold or more upon *Dpf2* deletion (Figures 4B and 4C). 21% of sites with decreased H3K27ac levels and 46% of sites with an increase in H3K27ac were bound by DPF2 in WT ESCs (Figure 4B) and located predominantly in enhancers (Figure S4B), indicating that *Dpf2* contributes to the regulation of H3K27ac at active enhancers in ESCs.

H3K4me1, H4-tetraAC, H3K9ac, and binding by Oct4 and Sox2 followed similar trends to H3K27ac at DPF2-occupied sites, and the repressive mark H3K27me3 exhibited an antithetical pattern (Figure 4D). P300 presence was dramatically affected at DPF2-bound sites with diminished H3K27ac levels in *Dpf2*^−/−^ ESCs but displayed very little change DPF2 sites with a gain of H3K27ac (Figure 4D). Thus, DPF2 controls the chromatin state and pluripotency TF binding at a subset of its target sites.

Given the extensive co-localization of DPF2 with OCT4 and the interaction between these two proteins, we investigated the effect of *Dpf2* loss on OCT4 binding in ESCs further. On average, OCT4 binding was not different between *Dpf2*^*fl/fl*^ and *Dpf2*^*−/−*^ cells at sites normally bound by DPF2 (Figure S4C). However, a more detailed analysis identified a significant decrease of OCT4 binding at 6,082 genomic locations in cells lacking *Dpf2* expression, with 27% of those exhibiting DPF2 binding in ESCs (Figures 4E and 4F) and an accompanying reduction in H3K27ac levels in *Dpf2*^−/−^ ESCs (Figure 4G). A much smaller number of sites displayed an increase in OCT4 binding in *Dpf2*^−/−^ ESCs (737), accompanied by an increase in H3K27ac, with many bound by DPF2 in ESCs (Figures 4E–4G). As seen in our H3K27ac analysis, DPF2-bound sites with changes in Oct4 levels were more enriched in enhancers than promoters (Figure S4B). Interestingly, DPF2 binding sites exhibiting loss of H3K27ac and OCT4 binding, respectively, were strongly enriched close to downregulated genes (Figure S4D). Conversely, DPF2-bound locations at genes with elevated expression upon *Dpf2* loss were enriched for changes in H3K27ac and OCT4 binding, but less strongly (Figure S4D). These results suggest that *Dpf2* can act as both a suppressor and activator of gene expression through the regulation of H3K27ac, P300, and OCT4 levels and that enhancers are key sites of its action.

The destabilization of OCT4 binding and reduction of H3K27ac upon loss of *Dpf2* is exemplified at enhancers within the *Bmp4* (Figure 4H), *Tbx3* (Figure 6A), *Gjb3*, Lama1,and *Nkx6-3* loci (Figures S4E–S4G). ChIP-qPCR confirmed this result for OCT4 binding at these genes and extended the findings to P300 (Figures S4H and S4I). Although the binding of SOX2 and NANOG at these loci also decreased (Figures S4J and S4K), we did not detect any interaction of the DPF2 protein with either SOX2 or NANOG in co-immunoprecipitation experiments (Figure S4L), suggesting that the loss of these TFs is due to OCT4 loss.

Even though overall BAF complex binding remained largely unaffected by the loss of *Dpf2* (Figure 3), 8% of the BRG1 sites exhibited a reduction in BRG1 occupancy and a concurrent reduction of H3K27ac, P300, and OCT4 (Figures 4I and 4J) and were located in enhancers enriched close to genes downregulated upon *Dpf2* loss (Figures S4B and S4D). Thus, at least for a number of sites, DPF2 loss associates with the de-stabilization of the entire BAF complex in addition to the reduction of OCT4 and P300. These sites include the examples described above (Figures 4H, S4E-S4G, and 6A). The targeting of new sites by BRG1 was negligible in Dpf2^−/−^ ESCs. The impaired binding of BRG1 at only a subset of sites may be explained by the existence of multiple BAF complexes in ESCs that consist of different core components. These results confirm a role of *Dpf2* in the recruitment of OCT4 and BRG1 at a subset of sites within ESC enhancers.

### DPF2 Occupancy Changes with Differentiation

**Figure 5:**
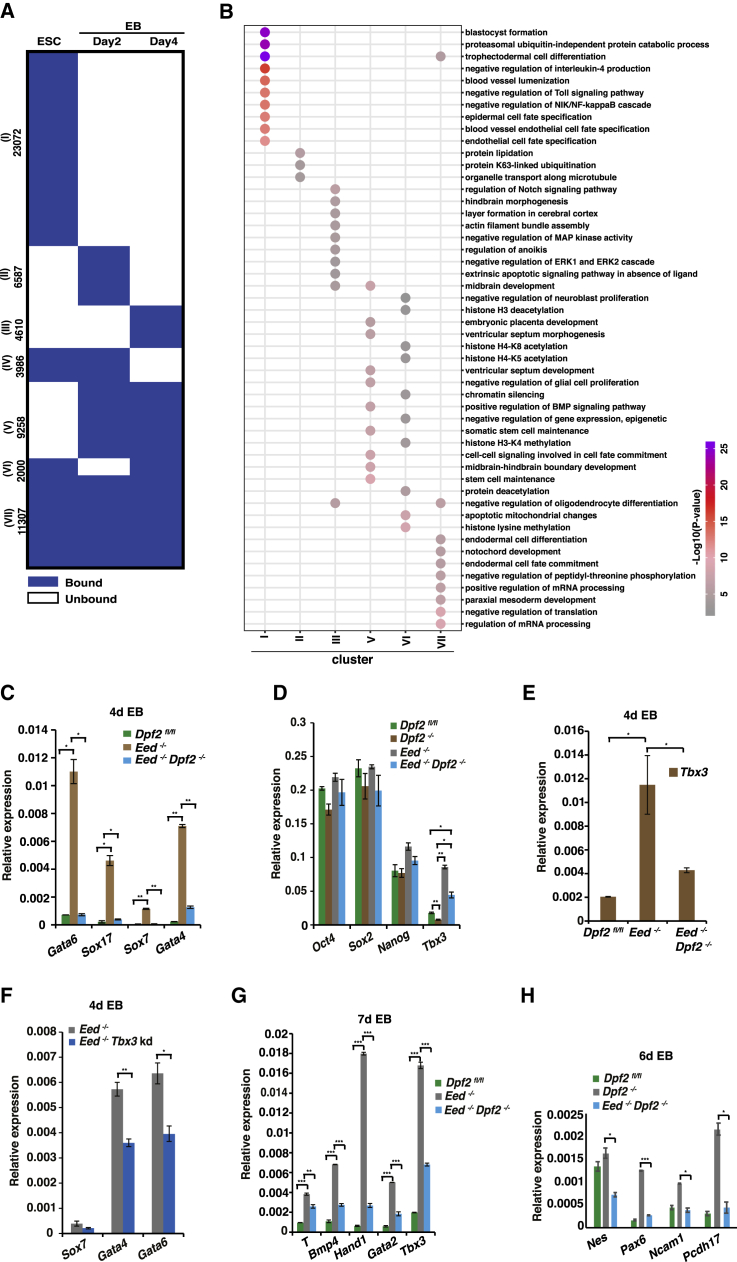
Regulation of Meso-endoderm Differentiation by *Dpf2* and *Eed* via *Tbx3* (A) Clustering of Dpf2 binding events in ESCs and day 2 and 4 EBs. The genome was divided into 500-bp bins and a bin called bound (blue) or unbound (white) based on the presence of a DPF2 peak. (B) GO analysis for enriched biological process for genes associated with DPF2 peaks from different clusters defined in (A), within ± 20 kb of the TSS. (C) Transcript levels of the indicated endoderm markers in day 4 EBs from *Dpf2*^*fl/fl*^, *Eed*^*−/−*^, and *Eed/Dpf2* double KO determined by qPCR. (D) As in (C), except for pluripotency-associated genes in *Dpf2*^*fl/fl*^, *Dpf2*^*−/−*^, *Eed*^*−/−*^, and *Dpf2* and *Eed* double KO ESCs. (E) As in (C), except for *Tbx3* in day 4 EBs from *Dpf2*^*fl/fl*^, *Eed*^*−/−*^, and *Eed/Dpf2* double KO ESCs. (F) As in (C), except for various endoderm markers in 4-day EBs induced from *Eed*^*−/−*^ and *Eed*^*−/−*^*/Tbx3* kd ESCs. (G) As in (C), except for various mesoderm markers and *Tbx3* in day 7 EBs from *Dpf2*^*fl/fl*^, *Eed*^*−/−*^, and *Eed/Dpf2* double KO ESCs. (H) As in (C), except for various neuroectoderm markers in day 6 EBs from *Dpf2*^*fl/fl*^, *Dpf2*^*−/−*^, and *Eed/Dpf2* double KO ESCs.

To gain mechanistic insights into the actions performed by DPF2 during differentiation, we performed ChIP-seq for DPF2 in EBs cultured for 2 or 4 days (Table S4). DPF2 bound a large number of new genomic locations as early as 2 days post-differentiation (clusters II, III, and V) (Figure 5A). Two-thirds of ESC binding events were lost upon differentiation (clusters I and IV) and one-third was maintained (clusters VI and VII) upon differentiation (Figure 5A). ESC-specific DPF2 binding sites were located in the vicinity of genes associated with blastocyst formation and trophectoderm differentiation (cluster I; Figure 5B), whereas newly bound sites in EB-neighbored genes associated with neuronal development (clusters III and V; Figure 5B), supporting a direct role of DPF2 in the regulation of neural ectoderm differentiation. Constitutively bound DPF2 sites were associated with endodermal and mesodermal as well as notochord development (cluster VII; Figure 5B) and contained binding events in the vicinity of the endodermal and mesodermal marker genes *Gata4*, *Gata6*, *Sox17*, and *T* (Figure S5A). Consistent with the maintenance of *Dpf2* binding, we did not observe a change in H3K27ac levels at these sites (Figure S5A). These data suggest that the gene expression changes in endo-mesodermal genes observed upon *Dpf2* deletion do not occur through a direct function of DPF2. Consistent with this idea, overexpression of *Dpf2* in ESCs did not lead to the upregulation of endo- and mesodermal markers (Figure S2F).

### *Dpf2* and *Eed* Control Meso-endoderm Differentiation by Opposingly Regulating *Tbx3*

Previous studies showed that the deletion of *Eed* increases the expression of endoderm and mesoderm markers (Boyer et al., 2006, Chamberlain et al., 2008, Leeb et al., 2010). Because we observed the downregulation of endo- and mesodermal genes in differentiating *Dpf2*^*−/−*^ ESCs (Figure 2), we hypothesized that endo- and mesoderm differentiation may be oppositely regulated by *Eed* and *Dpf2*. To test this idea, we deleted the *Eed* gene in *Dpf2*^*fl/fl*^ cells by targeting its second exon with an out-of-frame mutation leading to loss of the EED protein (Figures S5B–S5D). Immunostaining verified the loss of H3K27me3 in *Eed*^*−/−*^ ESCs (Boyer et al., 2006; Figure S5E). Subsequently, we induced differentiation of *Dpf2*^*fl/fl*^, *Eed*^*−/−*^, and *Eed*/*Dpf2* double KO ESCs by EB formation.

As expected, KO of *Eed* led to increased expression of the endodermal genes *Gata4*, *Gata6*, *Sox7*, and *Sox17* (Figures 5C and S5F) (Boyer et al., 2006). The expression of these genes was restored close to WT levels in *Dpf2* and *Eed* double KO EBs (Figures 5C and S5F), demonstrating that *Eed* and *Dpf2* regulate endoderm differentiation in an opposing manner.

Next we studied how *Eed* and *Dpf2* mechanistically regulate endoderm differentiation. Deletion of *Eed* did not dramatically affect the expression of *Oct4*, *Sox2*, and *Nanog* in ESCs but significantly increased *Tbx3* expression, contrary to the downregulation of *Tbx3* in *Dpf2*^*−/−*^ ESCs (Figure 5D). When we induced the deletion of *Dpf2* in *Eed*^*−/−*^ cells, *Tbx3* expression decreased toward WT levels (Figure 5D). We conclude that *Eed* and *Dpf2* oppositely regulate *Tbx3* expression in ESCs and during the onset of differentiation.

We further investigated whether the increase in *Tbx3* expression in the absence of *Eed* was responsible for the increase in endodermal gene expression. We found that *Tbx3* transcript levels correlated with the expression of *Gata4*, *Gata6*, *Sox7*, and *Sox17* during differentiation of *Dpf2*^*fl/fl*^, *Eed*^*−/−*^, and *Eed*/*Dpf2* double KO cells (Figures 5C, 5E, S5F, and S5G). Therefore, we induced EB formation from *Eed*^−/−^/*Tbx3* knockdown (kd) ESCs in which *Tbx3* transcripts were depleted by RNAi-mediated knockdown (Figure S5H). Expression of *Gata4*, *Gata6*, and *Sox7* decreased when *Tbx3* was depleted compared with *Eed*^*−/−*^ cells (Figure 5F), indicating that *Eed* controls endoderm differentiation, at least partially, via regulating *Tbx3* expression, and that the balance of *Dpf2* and *Eed* is critical for the regulation of *Tbx3*.

These conclusions extend to the regulation of mesoderm differentiation. Contrary to the downregulation of the mesoderm genes *T*, *Bmp4*, *Hand1*, and *Gata2* in differentiating *Dpf2*^*−/−*^ ESCs (Figure 2), the expression of these genes significantly increased in *Eed*^*−/−*^ EBs and was largely restored in *Dpf2* and *Eed* double KO EBs (Figure 5G). Similarly, the induced expression of the neural ectoderm markers *Nes*, *Pax6*, *Ncam1*, and *Pcdh17* observed in *Dpf2*^*−/−*^ EBs was restored to physiological levels in *Dpf2* and *Eed* double KO EBs (Figures 5H and S5I). Thus, in addition to endoderm differentiation, *Eed* and *Dpf2* oppositely regulate mesoderm and neural ectoderm differentiation.

### *Dpf2* and *Ezh2* Regulate Endo- and Mesoderm Differentiation by Regulating *Nanog*

Given that *Eed*, *Ezh2*, and *Suz12*, the core subunits of PRC2, have different effects on ESC differentiation (Boyer et al., 2006, Chamberlain et al., 2008, Leeb et al., 2010), we wanted to find out what the effects of *Ezh2* were in our system. We deleted the fifth exon of the *Ezh2* gene homozygously in *Dpf2* ^*fl/fl*^ cells, which resulted in loss of EZH2 at the protein level (Figure S6A), and induced EB formation of *Dpf2*^*fl/fl*^, *Ezh2*^*−/−*^, and *Ezh2* and *Dpf2* double KO ESCs. In contrast to the upregulation of endoderm markers in the absence of *Eed* (Figures 5C and S5F), KO of *Ezh2* led to repression of *Gata4*, *Gata6*, *Sox7*, and *Sox17* (Figures S6B and S6C). The expression of these genes was restored close to WT levels in *Dpf2* and *Ezh2* double KO EBs (Figures S6B and S6C), indicating that *Ezh2* and *Dpf2* regulate endoderm differentiation in an opposing manner but differently compared with *Eed*. *Ezh2* and *Dpf2* also opposingly regulated mesoderm differentiation (Figure S6D). The decreased expression of meso-endoderm genes in *Ezh2*^*−/−*^ EBs (Figures S6B–S6D) occurred despite an increase in *Tbx3* expression upon deletion of *Ezh2* (Figure S6E). Thus, *Eed* and *Ezh2* both repress *Tbx3* in ESCs but have rather distinct effects on meso-endoderm differentiation.

Consistent with previous reports (Shen et al., 2008, Villasante et al., 2011), *Nanog* was upregulated in *Ezh2*^*−/−*^ ESCs (Figure S6E). Because *Nanog* overexpression in ESCs is reported to repress endoderm and mesoderm lineages (Chambers and Smith, 2004), we postulated that the regulation of *Nanog* could explain the observed impairment of differentiation toward these lineages in *Ezh2*^*−/−*^ ESCs (Figures S6B and S6D). Indeed, the expression of endo- and mesodermal genes in *Ezh2*^*−/−*^ EBs increased when *Nanog* was knocked down by short hairpin RNA (shRNA) (Figures S6F and S6G). *Nanog* was downregulated when *Dpf2* was deleted in *Ezh2*^*−/−*^ ESCs (Figure S6E), which may restore the differentiation of *Ezh2* and *Dpf2* double KO ESCs to mesoderm and endoderm. Furthermore, we found that neural ectoderm genes were repressed in *Ezh2*^*−/−*^ EBs, which could be restored close to WT levels upon deletion of *Dpf2* (Figures S6H and S6I), demonstrating the opposing regulation of neural ectoderm differentiation by *Dpf2* and *Ezh2*.

Taken together, *Eed* and *Ezh2* hinder and promote meso-endoderm differentiation of ESCs, respectively. The PRC2 subunits achieve these opposing effects by acting through different downstream TFs (*Tbx3* versus *Nanog*). In contrast, during neural-ectoderm differentiation, both PRC2 subunits repress the program, suggesting that they may have the same downstream targets in this process.

### *Dpf2* and *Eed* Regulate *Tbx3* through Different Enhancers

**Figure 6:**
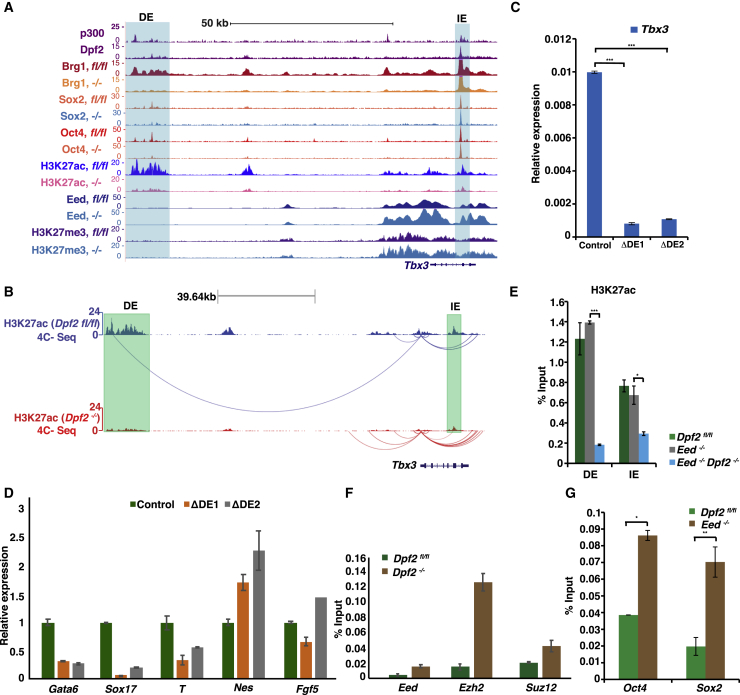
*Dpf2* and *Eed* Regulate *Tbx3* Expression by Controlling Histone Modifications and Accessibility of Pluripotency TFs at Different Enhancers (A) Genome browser view of ChIP-seq tracks for DPF2 and P300 in WT ESCs and BRG1, OCT4, SOX2, EED, H3K27ac, and H3K27me3 in *Dpf2*^*fl/fl*^ and *Dpf2*^*−/−*^ ESCs (indicated as fl/fl and −/−) at the *Tbx3* locus. The distal enhancer (DE) and intronic enhancer (IE) enhancers are highlighted. The values on the y axis represent fold enrichment over control. (B) Circular chromatin conformation capture (4C-seq) analysis of the *Tbx3* promoter. The arcs represent significant interactions from the *Tbx3* promoter viewpoint in *Dpf2*^*fl/fl*^ and *Dpf2*^*−/−*^ ESCs. The DE and IE are indicated. (C) Transcript levels of *Tbx3* in two DE KO ESC clones and control ESCs based on qPCR. (D) As in (C), except for the indicated lineage markers in day 6 EBs from WT and the two DE KO ESCs. (E) H3K27ac levels at *Tbx3* enhancers in *Dpf2*^*fl/fl*^, *Eed*^*−/−*^, and *Eed/Dpf2* double KO ESCs, determined by ChIP-qPCR. (F) As in (E), except for relative levels of EED, EZH2, and SUZ12 at the IE of the *Tbx3* gene in *Dpf2*^*fl/fl*^ and *Dpf2*^*−/−*^ ESCs. (G) As in (E), except for relative levels of OCT4 and SOX2 at the IE of the *Tbx3* gene in WT and *Eed*^*−/−*^ ESCs.

Because DPF2 predominantly binds active enhancers in ESCs, we speculated that *Dpf2* regulates *Tbx3* expression by regulating H3K27ac levels at its enhancers. Indeed, we found that *Dpf2* deletion in ESCs decreased H3K27ac of the previously described intronic enhancer (IE) (Buecker et al., 2014) and a distal enhancer (DE) located about 87 kb upstream of the TSS (Figure 6A). Interestingly, *Dpf2* deletion decreased OCT4 and SOX2 binding, but only at the DE and not at the IE (Figure 6A), suggesting that the DE may be critical for the regulation of *Tbx3* by *Dpf2* in ESCs. In agreement with this, with the circular chromosome conformation capture assay (4C) using the *Tbx3* promoter as a viewpoint, the *Tbx3* promoter was found in spatial proximity to the DE in *Dpf2*^*fl/fl*^ ESCs but to the IE in *Dpf2*^*−/−*^ ESCs (Figure 6B). Moreover, deletion of the DE (Figure S7A) significantly decreased *Tbx3* transcript levels (Figure 6C) and impaired meso-endoderm differentiation (Figure 6D). These data indicated that *Dpf2* regulates *Tbx3* expression mainly via the modulation of H3K27ac on the DE, which changes the access of OCT4 and other TFs or vice versa.

Considering how EED regulates *Tbx3*, we found that deletion of *Eed* did not affect the level of H3K27ac on either the IE or DE (Figure 6E). However, we identified a strong enrichment of H3K27me3 and EED at the IE and across the *Tbx3* gene in ESCs but not at the DE (Figure 6A). As expected, KO of *Eed* led to the loss of H3K27me3 at the IE (Figure S7B). Moreover, the deletion of *Dpf2* induced an increase of EED and H3K27me3 as well as the other two PRC2 subunits, EZH2 and SUZ12, at the IE and across the *Tbx3* gene body (Figures 6A and 6F) without a change in OCT4 and SOX2 binding at the IE (Figure 6A). Thus, *Dpf2* deletion in ESCs did not alter the accessibility of key pluripotency transcription factors but increased the access of PRC2 to the IE and across the *Tbx3* gene body. Deletion of *Dpf2* also led to decreased binding of the BRG1 on the DE region, which was not the case at the IE region because BRG1 binding was largely unaffected (Figure 6A). We also found that deletion of the IE in ESCs did not strongly affect the expression of *Tbx3* in ESCs (Figure S7C), suggesting that the IE does not contribute to the downregulation of *Tbx3* in *Dpf2*^*−/−*^ ESCs. However, OCT4 and SOX2 binding increased at the IE in *Eed*^*−/−*^ ESCs (Figure 6G), which may provide the mechanism for how the loss of *Eed* results in upregulation of *Tbx3*. Taken together, our data indicate that *Dpf2* regulates *Tbx3* by modulating H3K27ac and binding of pluripotency TFs at the DE, whereas *Eed* controls *Tbx3* through the regulation of H3K27me3 across the gene body and pluripotency factor access at the IE, which results in opposite outcomes regarding *Tbx3* expression.

### *Dpf2* and *Eed* Regulate Gene Expression Oppositely via Modulating Histone Modifications and Binding of Pluripotency TFs

Finally, we compared the transcription profiles of *Dpf2*^*−/−*^ and *Eed*^*−/−*^ ESCs to determine whether there are additional transcripts with similar trends as observed for *Tbx3*. We found 328 deregulated genes in both *Dpf2*^*−/−*^ and *Eed*^*−/−*^ ESCs (p < 0.05) (Figure S7D) and 2,482 shared target genes with both DPF2 and EED sites in their vicinity (Figure S7E). Intersecting the two sets of genes, we identified 144 genes directly regulated by *Dpf2* and *Eed* (Figure S7F). Among those, 34 genes were downregulated in *Dpf2*^*−/−*^ ESCs and upregulated in *Eed*^*−/−*^ ESCs, similar to the opposing regulation of *Tbx3* by *Dpf2* and *Eed* (Figure S7G). These genes included *Bmp4*, *Sox21*, and *Lama1* (Figures S7G and S7I) and were associated with embryonic development based on GO analysis (Figure S7H). Similar to *Tbx3*, the respective regulation of H3K27ac and H3K27me3 by *Dpf2* and *Eed* was observed for *Bmp4*, *Sox21*, and *Lama1* (Figures S7J and S7K). Moreover, the binding of OCT4 and SOX2 at these genes decreased in *Dpf2*^*−/−*^ ESCs (Figures S7L and S7M) but increased in *Eed*^*−/−*^ ESCs (Figures S7N and S7O). Thus, our study uncovered that *Dpf2* and *Eed* oppositely regulate a set of genes important for embryonic development by modulating the deposition of H3K27ac and H3K27me3 and altering the access of pluripotency TFs.

## Discussion

In this study, we revealed that the BAF subunit DPF2 is critical for ESC differentiation into mesoderm, endoderm, and neural ectoderm. Moreover, we show that meso-endoderm differentiation defects because of *Dpf2* deletion can be rescued by restoring the expression of *Tbx3* to normal levels. The differentiation defects of *Dpf2* KO ESCs are different from those described for other BAF components (Ho and Crabtree, 2010), in agreement with the notion that different subunits confer different functionalities. Importantly, this study defines a functional downstream target of the BAF complex in ESCs, for which maintenance of expression is important for ESC fate decisions.

Our study further revealed an opposing regulation of endoderm and mesoderm differentiation by *Dpf2* and *Eed*. This relationship was supported by the restored expression of endoderm and mesoderm marker genes in *Eed*/*Dpf2* double KO EBs. We postulate that *Dpf2* and *Eed* oppositely regulate endo- and mesoderm differentiation of ESCs via differential control of *Tbx3* expression.

**Figure 7:**
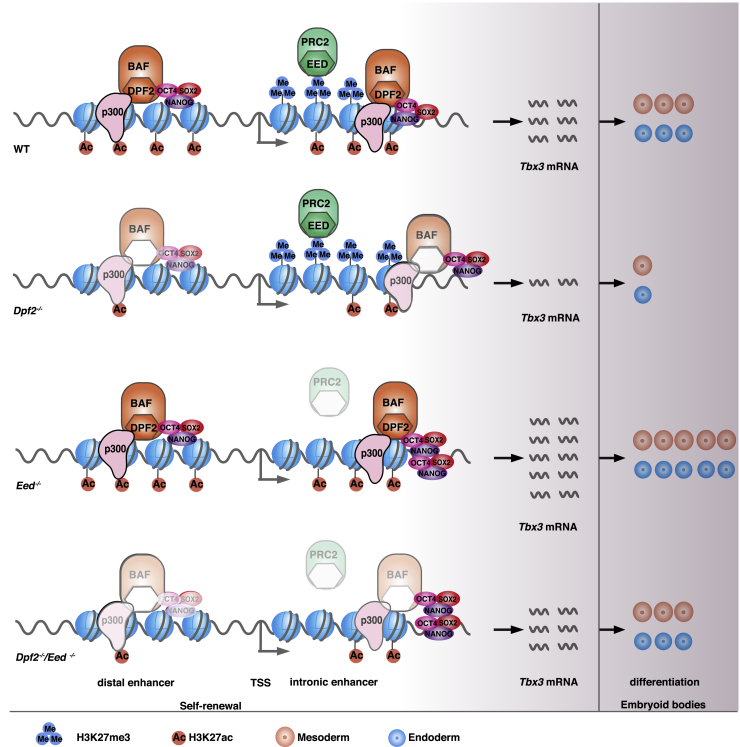
Model for the Regulation of ESC Differentiation by *Dpf2* and *Eed* via the Control of *Tbx3* Expression *Dpf2* and *Eed* regulate *Tbx3* expression via modulation of the H3K27ac level at the DE and the H3K27me3 level at the IE. Loss of *Dpf2* induces downregulation of *Tbx3* via a decrease of both H3K27ac and pluripotency TF binding on the DE, leading to impaired differentiation into meso-endoderm. In *Eed*^*−/−*^ ESCs, H3K27me3 levels decrease at the IE, whereas the binding of pluripotency TFs increases, resulting in *Tbx3* upregulation and enhanced differentiation to meso- and endoderm. In *Dpf2*^*−/−*^*/Eed*^*−/−*^ ESCs, *Tbx3* expression is restored to physiological ESC levels, as is the potential for differentiation into endo- and mesoderm.

An antagonistic role of polycomb and BAF complexes has been reported previously through competitive binding of these complexes at the same locus (Wilson et al., 2010, Ho et al., 2011, Kadoch et al., 2017). In contrast, our work shows that Eed and Dpf2 function in meso-endoderm differentiation via their respective interaction at different enhancers, the IE and DE, respectively, at the *Tbx3* locus, as summarized in Figure 7. Specifically, the loss of *Eed* diminished the enrichment of H3K27me3 over the *Tbx3* gene, including its IE, which increased the access of OCT4 and SOX2 to the IE, which likely leads to upregulation of *Tbx3*. The loss of *Dpf2* led to an increase of H3K27me3 deposition at the IE of *Tbx3* by increasing the access of PRC2, consistent with competitive binding between PRC2 and BAF complexes at the IE. Conversely, the loss of *Dpf2* significantly decreased the H3K27ac level and the access of OCT4 and SOX2 at the DE. The decrease in OCT4 binding could precede the drop of H3K27ac because the impaired physical interaction of DPF2 and OCT4 upon loss of *Dpf2* may destabilize OCT4 binding. Conversely, because P300 is known to acetylate histone H3K27 (Tee and Reinberg, 2014), another possible scenario for the decrease in H3K27ac is that loss of the direct interaction between DPF2 and P300 leads to the decrease in H3K27ac in *Dpf2*^*−/−*^ ESCs, which, in turn, may affect OCT4 binding. Regardless, the interaction between DPF2, P300, and OCT4 indicates a collaborative regulation of *Tbx3* via a chromatin remodeler, chromatin modifications, and critical TFs.

Our study also demonstrates that the opposing regulation of targets by *Dpf2* and *Eed* in ESCs is not limited to *Eed* but extends to the PRC2 subunit Ezh2. However, meso-endoderm markers were repressed in the absence of Ezh2, in contrast to their increase upon Eed deletion. We show that these differences in the differentiation defect are achieved through distinct downstream transcription factors because the opposing effect of *Dpf2* and *Ezh2* ensued mainly via differential regulation of *Nanog*.

*Brg1* is a core unit of BAF complex and is required for the self-renewal and pluripotency of ESCs (Ho and Crabtree, 2010). We confirmed the interaction of DPF2 and BRG1 by immunoprecipitation and showed that these proteins extensively co-localize in the genome. Although both *Brg1* and *Dpf2* positively regulate *Tbx3* expression in ESCs (Ho et al., 2011), *Dpf2* did not affect the expression of *Oct4*, which is upregulated upon acute deletion of *Brg1* (Bultman et al., 2000), indicating that *Dpf2* mediates specific functions of the BAF complex during embryonic development. As a core factor of the BAF complex, *Brg1* likely affects embryonic development by participating in various BAF complexes with different components (Ho and Crabtree, 2010, Panamarova et al., 2016). Consistent with this conclusion, loss of *Dpf2* does not dramatically alter the genome-wide binding of BRG1.

In summary, PRC2 and BAF complexes are important for the ESC differentiation and embryonic development (Ho and Crabtree, 2010). Our study uncovered unique mechanisms by which a specific BAF subunit and PRC2 subunits regulate genes important for the ESC differentiation.

## STAR★Methods

### Key Resources Table

**Table:** 

REAGENT or RESOURCE	SOURCE	IDENTIFIER
**Antibodies**		

Mouse monoclonal GAPDH	Abcam	ab9484
Rabbit polyclonal Requiem	Sigma	SAB4502621
Mouse monoclonal FLAG	Sigma	F1804
Rabbit polyclonal TBX3	Invitrogen	42-4800
Goat polyclonal OCT4	R&D systems	AF1759
Goat polyclonal SOX2	R&D systems	AF2018
Mouse monoclonal NESTIN	BD PharMingen	556309
Mouse monoclonal TUBB3	Promega	G712A
Rabbit polyclonal NANOG	Abcam	Ab80892
Mouse monoclonal P300	Active motif	61401
Mouse monoclonal STAT3	Cell Signaling Technology	9139
Rabbit monoclonal H3K27ac	Abcam	ab177178
Rabbit polyclonal H3K27me3	Millipore	07449
Rabbit polyclonal H3K4me3	Abcam	ab8580
Rabbit polyclonal H3K9ac	Abcam	ab4441
Mouse monoclonal H4-tetraAC	Active motif	39967
Polyclonal goat SOX17	R&D systems	AF1924
Rabbit polyclonal GATA4	Santa Cruz	sc-9053
Goat polyclonal GATA6	R&D systems	AF1700
Rabbit polyclonal SUZ12	Abcam	ab12073
Mouse monoclonal EZH2	BD PharMingen	612667
Rabbit polyclonal EED	Millipore	17-10034
Rabbit monoclonal BRG1	Abcam	ab110641
Mouse IgG	ThermoFisher	A-11032
Alex488-conjugated donkey anti-goat IgG	ThermoFisher	A-11055
Alex594-conjugated donkey anti-rat IgG	ThermoFisher	A-21209
Alex488-conjugated donkey anti-mouse IgG	ThermoFisher	A-21202
Alex594-conjugated donkey anti-rabbit IgG	ThermoFisher	A-21207
DAPI	ThermoFisher	D1306

**Bacterial and Virus Strains**		

DH5a Competent Cells	NEB	C-29871
One Shot TOP10 Chemically Competent *E. coli*	ThermoFisher	C404010

**Chemicals, Peptides, and Recombinant Proteins**		

LIF	Millipore	ESG1107
BMP4	R&D System	5020-BP-010
Zeocin	ThermoFisher	R25001
G418	ThermoFisher	11811031
Puromycin	ThermoFisher	A1113802
Protease inhibitors	Roche	4693159001
Benzonase	Sigma	E8263-5KU
2x Laemmli Sample Buffer	Bio-Rad	#1610737
3X FLAG peptide	Sigma	F4799-4MG
Trypsin-EDTA (0.25%), phenol red	ThermoFisher	25200056
Paraformaldehyde	Sigma	P6148-500G
Disuccinimidyl glutarate	ThermiFisher	20593
Triton X-100	Sigma	T8787-50ML
4-Hydroxytamoxifen	TOCRIS	3412
Tamoxifen	Sigma	T5648
Annexin-APC	BD PharMingen	550475
7-AAD	BD PharMingen	559925
SYBR Green PCR Master Mix	ThermoFisher	4309155
ABsolute QPCR Mix, ROX	ThermoFisher	AB1139A
SequalPrep Long PCR Kit with dNTPs	ThermoFisher	A10498
Expand Long Template PCR System	Roche	03321053103
Disuccinimidyl glutarate	ThermoFisher	20593
Proteinase K	ThermoFisher	25530049
T4 DNA ligase	NEB	M0202S
Csp6I	Thermo Fisher	FD0214
MboI	Thermo	FD0814
Vivaspin500 PES centrifugal filters	Vivascience	VS0102
KAPA HTP Library Preparation kit	Roche	07961901001

**Critical Commercial Assays**		

AP staining kit	Sigma	SCR004
SequalPrep Long PCR kit	Invitrogen	A10498
BCA Protein Assya Kit	Pierce	23227
ECL Plus	Amersham	RPN2133
RNeasy mini kit	QIAGEN	74104
TotalPrep RNA Amplification Kit	Ambion	AMIL1791
NucleoSpin gDNA Clean-Up kit	Macherey-Nagel	740230.10
SuperscriptIII reverse transcriptase	Invitrogen	18080093
Click-iT EdU Pacific Blue flow cytometry Assay Kit	Invitrogen	C10636
Pierce BCA Protein Assay Kit	Invitrogen	23225

**Deposited Data**		

ChIP-seq	This study	E-MTAB-6165
RNA-seq	This study	E-MTAB-6166
4C	This study	E-MTAB-6167

**Experimental Models: Cell Lines**		

E14 ESCs	This study	N/A
R26::CreERT2 - E14 ESCs	This study	N/A
R26::CreERT2 Dpf2 fl/fl E14 ESCs	This study	N/A
Eed −/− R26::CreERT2 Dpf2 fl/fl E14 ESCs	This study	N/A
Ezh2 −/− R26::CreERT2 Dpf2 fl/fl E14 ESCs	This study	N/A
Tbx3 DE KO ESCs	This study	N/A
Tbx3 IE KO ESCs	This study	N/A

**Experimental Models: Organisms/Strains**		

SCID mice	Hans Scholer’s Group	N/A

**Deposited Data**		

ChIP-seq data	This study	E-MTAB-6165 (ArrayExpress)
RNA-seq data	This study	E-MTAB-6166 (ArrayExpress)
4C data	This study	E-MTAB-6167 (ArrayExpress)
Proteomics data	This study	PXD011806 (ProteomeXchange)

**Oligonucleotides**		

*Oct4*	TaqMan	Mm00658129_gH
*Klf4*	TaqMan	Mm00516104_m1
*Nanog*	TaqMan	Mm02384862_g1
*Rex1*	TaqMan	Mm03053975_g1
*Nr0b1*	TaqMan	Mm00431729_m1
*Klf2*	TaqMan	Mm01244979_g1
*Klf5*	TaqMan	Mm00456521_m1
*Gapdh*	TaqMan	4352339E
*Tbx3*	TaqMan	Mm01195726_m1
*GATA6*	TaqMan	Mm00802636_m1
*GATA4*	TaqMan	Mm00484689_m1
*Sox17*	TaqMan	Mm00488363_m1
*FGF5*	TaqMan	Mm00438918_m1
*Sox1*	TaqMan	Mm00486299_s1
*Pax6*	TaqMan	Mm00443072_m1
*Brachyury*	TaqMan	Mm01318252_m1
*Tubb3*	TaqMan	Mm00727586_s1
*Dpf2*	TaqMan	Mm00599980_m1
*Nestin*	TaqMan	Mm00450205_m1
*Pdgfra*	TaqMan	Mm00440701_m1
*GATA2*	TaqMan	Mm00492301_m1
*Bmp4*	TaqMan	Mm00432087_m1
*Hand1*	TaqMan	Mm00433931_m1
*Sox21*	TaqMan	Mm00844350_s1
*Gjb3*	TaqMan	Mm00433647_m1
*Lama1*	TaqMan	Mm01226102_m1
*Ncam1*	TaqMan	Mm01149710_m1
*Pcdh17*	TaqMan	Mm00977568_m1
See Table S5 for qPCR and gRNA sequences	This Study	N/A

**Recombinant DNA**		

Dpf2 targeting vector	EUCOMM resource	N/A
C-FTAP-tag Dpf2 knockin vector	This study	N/A
pPyCAG-*Dpf2*-IZ	Hitoshi Niwa	N/A
pPyCAG-*Tbx3*-IN	Hitoshi Niwa	N/A
pCAGGs-FlpE	This study	N/A
Nanog shRNA	Wu Qiang	N/A
Tbx3 shRNA	April Kartikasari	N/A

**Reagent or Resource**		

GMEM	Sigma-Aldrich	G2549
FCS	GIBCO	10439024
Non-essential amino acid	GIBCO	11140050
Sodium pyruvate	GIBCO	11360070
2-mercaptoethanol	GIBCO	21985023
L-glutamine	GIBCO	25030081
TCEP	Sigma	75259
Iodoacetamide	Sigma	I6125
colloidal Coomassie	Sigma	B2025
AggreWell 400 plates	STEMCELL Technoligies	34421
Dynabeads Protein G	Invitrogen	10003D
4-12% Bis-Tris Novex gel	Invitrogen	NP0321BOX
PVDF membranes	Biorad	1620177

**Software and Algorithms**		

ChromHMM v1.1.0	Chronis et al., 2017	N/A
Bowtie2 v2.2.1	Langmead et al., 2009	N/A
Cufflinks v2.2.1	Trapnell et al., 2010	N/A
MACS2 v20140616	Zhang et al., 2008	N/A
BedTools v2.27	Quinlan and Hall, 2010	N/A
HOMER v4.9.0	Heinz et al., 2010	N/A
Metascape	http://metascape.org	N/A
Proteome Discover 1.4	Thermofisher	N/A
Tophat version 2.0.13	Trapnell et al., 2009	N/A
Mascot 2.5	Matrix Science	N/A

### Contact for Reagent And Resource Sharing

Further information and requests for reagents may be directed to and will be fulfilled by the Lead Contact, Wensheng Zhang (zhangwensheng@suda.edu.cn).

### Experimental Model and Subject Details

#### Cell culture

E14 ESCs were cultured in GMEM (Sigma) supplemented with 10% FCS, 1 × NEAA, 1 mM sodium pyruvate, 0.1 mM 2-mercaptoethanol, 2 mM L-glutamine, and LIF (Millipore) on gelatin coated plates, or cultured in N2B27 medium with BMP4 (10ng/ml) and LIF.

### Method Details

#### Colony formation assay

For colony formation assays, dissociated cells with trypsin were plated at about 1,000 cells per 10cm plate. ESCs were cultured for 7 days and stained for alkaline phosphatase using the AP staining kit (Sigma). We scored colonies with ∼90% AP-positive cells as un-differentiated, colonies with ∼5% AP-positive staining cells as differentiated, and colonies of intermediate AP-positive cell number as partially differentiated.

#### Teratoma formation assay

5 × 10^6^ cells were injected subcutaneously into the flank of SCID mice. After 4-5 weeks, teratomas were isolated, transferred into Bouin’s fixative overnight and subjected to histological examination with H&E staining based on standard protocols. All tissues were examined by a board-certified anatomic pathologist (M.P.), blinded to the genotype of the ESCs.

#### Generation of a conditional knockout of Dpf2 in ESCs

The *Dpf2* targeting vector was linearized and electroporated into R26::CreERT2 E14 cells to generate heterozygous ESC lines after G418 selection. Heterozygous ESC clones were transiently transfected with a FLP recombinase encoding plasmid (pCAGGs-FlpE), converting the initial knockout allele (*Dpf2*^*+/−*^, lacZ positive, G418 resistant) into a “wild-type” (WT) allele with two loxP sites flanking exon 4 (floxed allele) (*Dpf2*^*fl/+*^, reverted WT (rWT), lacZ negative, G418 sensitive). Multiple independent rWT ESC clones were then electroporated with the original *Dpf2* knockout vector and again selected with G418. Targeting of the second WT allele was confirmed by the presence of both the rWT allele and the second knockout allele through long-range PCR reactions (SequalPrep Long PCR kit, Invitrogen). Selected heterozygous ESC lines were converted to the conditional *Dpf2*^*fl/fl*^ state by transiently transfecting FlpE.

#### Generation of Eed and Ezh2 knockout ESC clones

2ug of gRNA and 2ug of Cas9 plasmids were electroporated to ESCs. After 7 days’ selection with 175ug/ml of G418, colonies were picked up for genotyping and confirmed by Sanger sequencing.

#### Co-Immunoprecipitation (Co-IP) and Western Blotting

Cells were lysed in lysis buffer (1% NP40; 50mM Tris-HCl, pH7.4; 150mM NaCl; 1mM EDTA with protease inhibitors (Roche)). Protein concentrations were determined using the BCA Protein Assay Kit (Pierce). For immunoprecipitation, cell lysates were incubated with the indicated antibodies for 1 hour. Protein G-associated Dynabeads^®^ (Thermo Fisher) were added at 4°C overnight. After washing three times with lysis buffer, 1X protein SDS loading buffer (Bio-Rad) was added and boiled for 5 minutes. The supernatant was cooled on ice for 5 minutes before loading on the gel for immunoblotting. Proteins were fractionated on a 4%–12% Bis-Tris Novex gel (Invitrogen), electroblotted onto PVDF membranes, and membranes probed sequentially with respective antibodies. Blots were incubated with secondary antibodies and developed with ECL Plus (Amersham).

#### Affinity purification of the DPF2 complex

Formaldehyde-crosslinked ESCs expressing DPF2-FTAP were used for affinity purification of DPF2, and an ESC line expressing a beta-gal-FTAP fusion protein (Bode et al., 2016) was used as a control. Whole cell extracts were prepared using a high salt lysis buffer (450 mM NaCl, 0.2% Nonidet P-40) as previously described (Pardo et al., 2010), with several modifications. Briefly, cells were crosslinked with 1% formaldehyde for 10 minutes at room temperature; after a 10 min incubation of cells in lysis buffer on ice, 1 ul/mL of benzonase (99% purity, Sigma) was added and the cell suspension was incubated at 37°C for 15 min. The lysate was then cleared by centrifugation at 16,100 r*cf.* for 15 min at 4°C. FLAG affinity purification was essentially performed as previously described. Anti-FLAG Dynal beads were prepared by crosslinking M2 FLAG antibody (Sigma) to Protein G-Dynal beads (Invitrogen) in accordance with the manufacturer’s instructions. Whole-cell extracts were incubated with anti-FLAG M2 Dynal beads in buffer containing 150 mM NaCl and 0.1% NP-40 for 90 min at 4°C. Beads were washed three times with RIPA buffer, then 3 times with RIPA buffer containing 450 mM NaCl, and once with elution buffer (10 mM Tris-HCl pH 8, 150 mM NaCl, 0.02% Nonidet P-40). Proteins were eluted in elution buffer containing 200 μg/mL 3X FLAG peptide (Sigma). Eluates were concentrated in Vivaspin500 PES centrifugal filters (10 kDa cut-off, Vivascience), reduced with 5 mM TCEP (Sigma), and alkylated with 10 mM iodoacetamide (Sigma). Samples were fractionated by polyacrylamide gel electrophoresis using Novex NuPAGE Bis-Tris 4%–12% gels (Invitrogen) and stained with colloidal Coomassie (Sigma) as previously described (Pardo et al., 2010). Full gel lanes were sliced in 7-24 bands, gel pieces were de-stained completely and digested with trypsin (sequencing grade, Roche). Peptides were extracted using 0.5% formic acid-50% acetonitrile and dried in a Speed Vac (Thermo Fisher Scientific).

#### Mass spectrometry

Peptides were re-dissolved in 0.5% formic acid and analyzed on an Ultimate 3000 RSLCnano System (Dionex) coupled to an LTQ FT Ultra (Thermo Fisher Scientific) hybrid or Orbitrap Velos mass spectrometer equipped with a nanospray source. The peptides were first loaded and desalted on a PepMap C18 trap column (0.1 mm id x 20 mm, 5μm), then separated on a PepMap 75 μm id x 25 cm column (5μm) over a 60 min linear gradient of 4 – 42% B / 90 min cycle time when coupled with FT Ultra, or 15 cm column over a 30 min linear gradient of 4 – 40% B / 60 min cycle time when coupled with Orbitrap Velos, where B is 80% CH3CN/0.1% Formic Acid. The LTQ FT Ultra was operated in the “top 5” data-dependent acquisition mode with the preview mode of FT master scan enabled. The FT full scan was set at m/z 380 – 1800 with the resolution at 100,000 at m/z 400 and AGC at 1x106 with a maximum injection time at 500 msec. The five most abundant multiply-charged precursor ions, with a minimal signal above 1000 counts, were dynamically selected for CID fragmentation (MS/MS) in the LTQ ion trap, with the AGC set at 1x104 with the maximum injection time at 200 msec. The dynamic exclusion was set at ± 20 ppm for 45 s. For analysis on the LTQ Orbitrap Velos, the mass spectrometer was operated in the “top 10” data-dependent acquisition mode with preview mode of FT master scan enabled. The Orbitrap full scan was set at m/z 380 – 1500 with the resolution at 100,000 at m/z 400 and AGC at 1x106 with a maximum injection time at 200 msec. The 10 most abundant multiply-charged precursor ions, with a minimal signal above 2000 counts, were dynamically selected for CID fragmentation (MS/MS) in the LTQ ion trap, with the AGC set at 5000 with the maximum injection time at 100 msec. The dynamic exclusion was set at ± 10 ppm for 60 s.

#### Immunofluorescence staining

Cells were fixed in 4% paraformaldehyde for 10 minutes at room temperature, blocked, and permeabilized with 3% serum in PBS with 0.3% Triton X-100 and then incubated with the indicated antibodies at 4°C overnight. After washing, cells were incubated with Alex594-conjugated goat anti-mouse IgG (ThermoFisher, A-11032), Alex488-conjugated donkey anti-goat IgG (ThermoFisher, A-11055) or Alex594-conjugated donkey anti-rat IgG (ThermoFisher, A-21209) and counter-stained with DAPI to detect nuclei.

#### Quantitative RT-PCR

Total RNA was isolated with RNeasy mini kit (QIAGEN). cDNA was synthesized with Superscript III reverse transcriptase (Invitrogen). Real-time PCR was performed with TaqMan Gene Expression Assay (Applied Biosystems). Gene expression was determined relative to Gapdh transcript levels. Standard deviation was calculated from PCR triplicates. Error bars give the SD of three technical qPCR replicates from a representative experiment.

#### Apoptosis Assays

*Dpf2*^*fl/fl*^ and WT ESCs were treated with ethanol or 0.65 μM of 4-Hydroxytamoxifen (4-OHT) for 96 hours, subsequently, cells were harvested with trypsin and washed with PBS. ESCs were then stained with Annexin-APC (BD Biosciences) and 7-AAD (BD Biosciences) according to the manufacturer’s instructions (BD Biosciences) and analyzed by flow cytometry. Cells were gated and analyzed for annexin V and 7-AAD. High level of annexin V and low levels of 7-aminoactinomycin D (7-AAD) show early apoptosis in cells, whereas high levels of both annexin V and 7-AAD indicate a late stage of apoptosis. Cells were considered healthy if the levels of both annexin V and 7-ADD were low.

#### Cell Cycle assay

*Dpf2*^*fl/fl*^ and WT E14 cells were cultured in the presence or absence of 4-OHT condition for five days before the cell cycle assay was performed. ESCs were trypsinized, re-plated and cultured in standard lif/serum ESC medium with 10uM of EdU. After incubation for 1 to 3 hours, cells were harvested for the cell cycle assay using the Click-iT^@^ EdU Pacific Blue flow cytometry Assay Kit (Invitrogen).

#### Chromatin Immunoprecipitation (ChIP) coupled with Quantitative Real-Time PCR (ChIP-qPCR)

ChIP-qPCR was performed as previously described (Chen et al., 2013). Briefly, ChIP experiment were performed as described below in the ChIP-seq section. After purification of the immunoprecipitated DNA, 1 μL was used per qPCR reaction. Bound regions were detected by using paired primers given in Table S5. Real -time PCR was run using SYBR Green Mix (2x) from Applied Biosystems. Each reaction contained 10 μL 2x SYBR Green Mix, 1 μL 10 μM Primer mix, 8 μL H2O and 1 μL immunoprecipitated DNA. The program was used as follows: 98°C 5 minutes, (98°C 20 s, 60°C 30 s, 72°C 20 s) X 40. Quantitative PCR was performed at least in duplicate, from at least two independent experiments, and data were normalized to input values and calculated as percent input recovery using the ΔΔCt method.

#### ChIP-seq

ChIP was typically performed in *Dpf2*^*fl/fl*^ and *Dpf2*^*−/−*^ ESC lines, except for DPF2, which were done in C-FTAP tag Dpf2 knockin ESCs and and EBs formed for 2 and 4 days respectively. Transcription factor and epigenetic regulator occupancy data generated in this study were acquired using ChIP after crosslinking cells. Briefly, cells were grown to a final concentration of 5x10^7^ cells for each ChIP-seq experiment. To stabilize DPF2, BRG1, P300, EED, Oct4 and Sox2 on chromatin, cells were treated with 2 mM disuccinimidyl glutarate (DSG) for 10 minutes prior to formaldehyde crosslinking. For all other targets (H3K4me3, H3K4me1, H3K27ac, H3K9ac, H4tetra-ac, H3K27me3), cells were cross-linked at room temperature by the addition of formaldehyde to 1% final concentration for 10 minutes and quenched with 0.125 M final concentration of glycine. Cross-linked cells were re-suspended in sonication buffer (50mM HEPES-KOH pH 7.5, 140mM NaCl, 1mM EDTA, 1% Triton X-100, 0.1% Na-deoxycholate, 0.1% SDS) and sonicated using a Diagenode Bioruptor for three 10-minute rounds using pulsing settings (30 s ON; 1 min OFF). 10 μg of sonicated chromatin was then incubated overnight at 4°C with 5 μg of Flag antibody conjugated to magnetic beads. Following the IP, beads were washed twice with RIPA buffer (50mM Tris-HCl pH8, 150 mM NaCl, 2mM EDTA, 1% NP-40, 0.1% Na-deocycholate, 0.1% SDS), low salt buffer (20mM Tris pH 8.1, 150mM NaCl, 2mM EDTA, 1% Triton X-100, 0.1% SDS), high salt buffer (20mM Tris pH 8.1, 500mM NaCl, 2mM EDTA, 1% Triton X-100, 0.1% SDS), LiCl buffer (10mM Tris pH 8.1, 250mM LiCl, 1mM EDTA, 1% Na-deoxycholate, 1% NP-40), and 1X TE. Finally, DNA was extracted by reverse crosslinking at 60°C overnight with proteinase K (20ug/μL) and 1% SDS followed by phenol:chloroform:isoamyl alcohol purification and ethanol precipitation. All protocols for Illumina/Solexa sequencing library preparation, sequencing, and quality control were performed as recommended by Illumina, with the minor modification of limiting the PCR amplification step to 10 cycles and sequenced using single-end 50 bp reactions on a HiSeq4000.

#### RNA-seq

Total RNA was purified by RNeasy Minikit (QIAGEN) according to the manufacturer’s manual. RNA concentration was determined using Nanodrop, and 500 ng of total RNA was used for library construction using the KAPA Stranded mRNA-seq Kit. Sequencing was performed on Illumina HiSeq 2500 machines with 125 bp pair-end mode.

#### Microarray analysis

*Dpf2*^*fl/fl*^ ESCs were treated with ethanol or 4-OHT for 48 hours before the induction of EBs formation. cRNA samples for global gene expression analyses were prepared with the linear TotalPrep RNA Amplification Kit (Ambion). Hybridizations on mouse-8 V2 chips (Illumina) were carried out as recommended by the manufacturer.

#### Circularized Chromosome Conformation Capture (4C-seq)

4C experiments were performed on *Dpf2*^*fl/fl*^ and *Dpf2*^*−/−*^ ESCs using two 4-cutter DNA restriction enzymes. The experiments were carried out in two technical replicates, where around 10 million cells per biological sample were used. Cells were cross-linked with 1% formaldehyde for 10 minutes followed by cell lysis and nuclei isolation. The resulting nuclei were enzymatically digested with 1 μL/μg of fast digest MboI (Thermo Fisher Scientific) for 4.5 hours at 37°C. Subsequently, the material was ligated with 12 Weiss units of T4 DNA ligase (New England Biolabs) for 4.5 hours at 16°C. The ligation products were then purified using phenol-chloroform-isoamyl alcohol followed by precipitation with ethanol. Subsequently the purified DNA was subjected to secondary digestion with 1 μL/μg of Csp6I (Thermo Fisher Scientific) for 2 hours at 37°C. The digested DNA was then ligated with 12 Weiss units of T4 DNA ligase (New England Biolabs) for 4.5 hours at 16°C to generate circularized chimeric DNA, which was ethanol precipitated and cleaned using NucleoSpin gDNA Clean-Up (Macherey-Nagel) silica-membrane columns. The reading primer was designed for a 229bp region downstream of the TSS of *Tbx3* gene and the amplification was carried out using Expand Long Template PCR System (Roche). The sequence of the reading primer was 5′-TTGCACCCGTCTTCTTGATC-3′. The amplification reactions consisted of 100ng of DNA primed with 35 picomoles of each, forward and reverse primers, and 200 μM dNTPs. 1.75 U of a *Taq* and *Tgo* polymerase blend catalyzed the reaction. Thermal cycling was performed in GeneAmp PCR System 9700 (Applied Biosystems) following the protocol of initial denaturation at 94°C for 2 min, 29 cycles of 94°C for 10 s, 55°C for 1 min, 68°C for 3 min, and ended by the final elongation at 68°C for 5 min. Amplification products were directly used for DNA libraries preparation for Illumina single index, paired end sequencing using NextSeq 500 system (Illumina Inc.). The DNA libraries were prepared using KAPA HTP Library Preparation kit for Illumina Platforms following manufacturer’s instructions entailing the end-repair of the amplified fragments, as well as A-tailing and TruSeq LT (Illumina Inc.) adaptor ligation. Prepared DNA libraries were purified and subjected for sequencing. 4C-seq was done in replicates.

### Quantification and Statistical Analysis

#### Statistics

RNA-seq and ChIP-seq raw data are discrete count-based data, which follow negative binomial or Poisson distribution (Marioni et al., 2008, Love et al., 2014), and no additional methods were used to determine whether the data met assumptions of the statistical approach. The experiments in Figures 2C and 2D and S1A were performed once with two independent Dpf2 mutant ESC clones; all other experiments were performed three times or more. In all Figures, n = number of biological replicates or number of clones. All q-PCR data represent the mean of three technical replicates. All error bars represent standard deviation (SD). The Student’s t test (unpaired, two-sided) was used to determine the significance of changes in the qPCR using Microsoft Excel. ^∗^ indicates p < 0.05, ^∗∗^ p < 0.01, ^∗∗∗^ < 0.001. For all other statistics tests, they were specified and performed using indicated bioinformatics software described below in this section.

#### Mass spectrometry analysis

Raw files were processed with the Proteome Discover 1.4 pipeline (Thermo Fisher Scientific). Database searches were performed with Mascot 2.5 (Matrix Science) against the mouse SwissProt database (v. January 2015). The search parameters were: trypsin/P with a maximum of 2 missed cleavages, 10 ppm mass tolerance for MS, 0.5 Da tolerance for MS/MS, with variable modifications of carbamidomethyl (C), N-acetylation (protein N-term), deamidation (NQ), formyl (N-term), oxidation (M), and Gln- > pyro-Glu (N-term Q). Database search results were refined through processing with Mascot Percolator. Protein identification required at least one high-confidence peptide (FDR < 1%). External contaminants (keratins, albumin, casein, immunoglobulins and TEV protease) were removed before further analysis. Protein lists from DPF2-FTAP experiments were compared to beta-gal-FTAP controls. High confidence DPF2 interactors were identified as those solely in DPF2-FTAP experiments, or with at least 3 times more sequences in DPF2-FTAP than in control experiments. We report these high confidence interactors identified by more than one peptide in at least one replicate in Table S1.

#### Data analysis

For ChIP-seq data, reads were mapped to the mouse mm9 reference genome using bowtie version 1.1.1 (Langmead et al., 2009) with -m 1 flag, which only allows uniquely mapped reads to be considered in the downstream analysis. Peaks were called using Macs2 (version 2.1.0.20151222)_(Zhang et al., 2008) with–nomodel–extsize 200 -q 0.01 flags. Two biological replicates were performed per experiment, and only peaks that were present in both replicates were considered. *De novo* Motif discovery was done using findMotifsGenome.pl from the HOMER suite (Heinz et al., 2010) (version 4.8) on the narrowPeak files returned from Macs2. For the analysis of peak overlap between different factors, intersectBed from the bedtools suite (Quinlan and Hall, 2010) was used, and overlapping peaks were defined as two peaks with at least 1 bp overlap. For differential levels of H3K27ac and differential binding by OCT4 or BRG1 in *Dpf2*^*fl/fl*^ and *Dpf2*^*−/−*^ ESCs, a union peak set was created first between wild-type and knockout ChIP-seq samples using mergeBed from the bedtools suite. Briefly, the narrowPeak files from wild-type and knockout ESCs were merged if they had at least 1 bp overlap. The number of mapped reads from each condition was counted on each of the union peaks using coverageBed from the bedtools suite. The number of reads of each union peak was normalized by the sequencing depth of different samples. For each union peak of OCT4, BRG1 and H3K27ac, we assigned differentially bound genomic locations if at least 2-fold difference between the wild-type and knockout samples was observed.

For RNA-seq data analysis, reads were mapped to the mouse mm9 reference genome with Tophat (Trapnell et al., 2009) version 2.0.13 and supplied with gene annotation from RefSeq. Gene expression was quantified by cuffquant, and differential gene expression test was performed using cuffdiff. Both cuffquant and cuffdiff were from the cufflinks package (Trapnell et al., 2010) (version 2.2.1).

Microarray data analysis was done in BeadStudio and MS Excel.

#### GO analysis

GO analysis for enriched biological processes was performed using Metascape (http://metascape.org) to find significantly enriched terms (P value ≤ 0.01).

#### ChromHMM ESC states, TF enrichments and data visualization

Chromatin state segmentations for the ESCs were obtained from (Chronis et al., 2017). To calculate the enrichment of binding events in distinct chromatin states, we utilized the ChromHMM OverlapEnrichment function as previously described (Chronis et al., 2017). The enrichment score was calculated as the ratio between observed and expected overlap between the binding event of interest and chromatin state after accounting their relative size and the size of the mouse genome.

To produce the heatmaps in Figures 3F/H, 4C/G/I and S3D/E we aligned the given feature (such as peaks of DPF2, BRG1 or EED) at their summit and tiled the flanking up- and downstream regions within ± 2kb in 100bp bins. For each location, we calculated RPKM values over all 100bp bins by using the number of sequencing reads that overlapped with each bin after extension by 50bp in the direction of the alignment. To normalize to the input control, we computed at each corresponding bin a log_2_ input-normalized RPKM value as log_2_(RPKM_FOREGROUND_) - log_2_(RPKM_Input_). For visualization in figures, each 100 bp bin was displayed with JavaTreeview. All metaplots were produced by computing the average input-normalized RPKM value for each 100bp bin across all locations in the given set. In Figure S4D, the fold-enrichment of Dpf2 in the vicinity (+/− 20Kb of the TSS) of up- and downregulated genes at sites exhibiting H3K27ac, Oct4 or Brg1 binding gain or reduction was calculated with the following formulas:(% Upregulated Dpf2 bound genes within region of interest) / (%All Dpf2 bound genes within regions of interest) and (% Downregulated Dpf2 bound genes within region of interest)/ (%All Dpf2 bound genes within regions of interest).

#### TF clustering and pairwise comparisons with optimal leaf ordering

K-means clustering was employed to identify constitutive and stage specific binding of DPF2 in ESCs and EBs in Figures 5A. To define these TF clusters, the genome was tiled into 500bp windows and the presence of TF peaks in each bin was determined. This procedure resulted in a vector of binary data for each TF reflecting its absence or presence within 500bp windows across the genome. The windows represented by these vectors were then clustered using R’s k-means function applying the Hartigan-Wong method to obtain groups of windows exhibiting common combinatorial binding patterns across the genome.

In Figure 3E we applied complete linkage hierarchical clustering with optimal leaf ordering to cluster the enrichments of all pairs of TFs (Bar-Joseph et al., 2001). The pairwise enrichments at base-pair resolution were calculated as the observed overlap divided by the expected overlap based on the binomial background model that treats both transcription factors as independent:

$$\mathrm{Enrichment}\left(TF_{A},TF_{B}\right)=\min\left(\frac{100+TF_{A}\cap TF_{B}}{100+TF_{A}*TF_{B}/G},500\right)$$

- where the numerator is the size of the overlap between peaks of TF_A_ and TF_B_ and the denominator is the product between the total number of bp occupied by peaks of TF_A_ and TF_B_ divided by the size of the genome (G). The maximum enrichment was set to 500. TF datasets for TFs not generated at this study were obtained from Chronis et al., GEO: GSE90895.

#### Circularized Chromosome Conformation Capture (4C) analysis

Resulting data were mapped with Bowtie 2 after trimming primer sequences. Duplicates and low quality reads were discarded. Counts for restriction enzyme fragments were generated using Bioconductor package FourCSeq (Klein et al., 2015). Only the interactions that were supported by at least 16 read pairs (corresponding to FDR = 0.1) in both replicates were taken forward.

### Data and Software Availability

The accession numbers for ChIP-seq, RNA-seq and 4C data are E-MTAB-6165, E-MTAB-6166 and E-MTAB-6167, respectively on ArrayExpress. The proteomics data are available via ProteomeXchange with identifier PXD011806.

## Supplemental Information

Document S1. Figures S1–S7 and Tables S3 and S5

Table S1. List of Proteins Identified from DPF2 Cross-Linking Affinity Purification Coupled to MS Experiments, Related to Figure 1 # Peptides indicates number of unique peptide sequences matched; # PSM indicates number of peptide spectrum matches.

Table S2. Differentially Expressed Genes between *Dpf2*^*fl/fl*^ and *Dpf2*^−/−^ ESCs, Related to Figures 2 and S2

Table S4. Locations of *Dpf2* Binding Events in ESCs and Day 2 and Day 4 Ebs, Related to Figure 5

Document S2. Article plus Supplemental Information
