# Supplementary material for: The BAF and PRC2 Complex Subunits Dpf2 and Eed Antagonistically Converge on Tbx3 to Control ESC Differentiation
Source: Cell Stem Cell. 2019 Jan 3;24(1):138–152.e8. doi: 10.1016/j.stem.2018.12.001 (PMC6486830; doi:10.1016/j.stem.2018.12.001)
Supplement: Document S1. Figures S1–S7 and Tables S3 and S5 [file mmc1.pdf]

Cell Stem Cell, Volume *24*

Supplemental Information

The BAF and PRC2 Complex Subunits Dpf2 and Eed Antagonistically Converge on Tbx3 to Control ESC Differentiation

Wensheng Zhang, Constantinos Chronis, Xi Chen, Heyao Zhang, Rapolas Spalinskas, Mercedes Pardo, Liangliang Chen, Guangming Wu, Zhexin Zhu, Yong Yu, Lu Yu, Jyoti Choudhary, Jennifer Nichols, Mana M. Parast, Boris Greber, Pelin Sahlén, and Kathrin Plath

Supplementary Data Figure 1 (related to Figure 1)

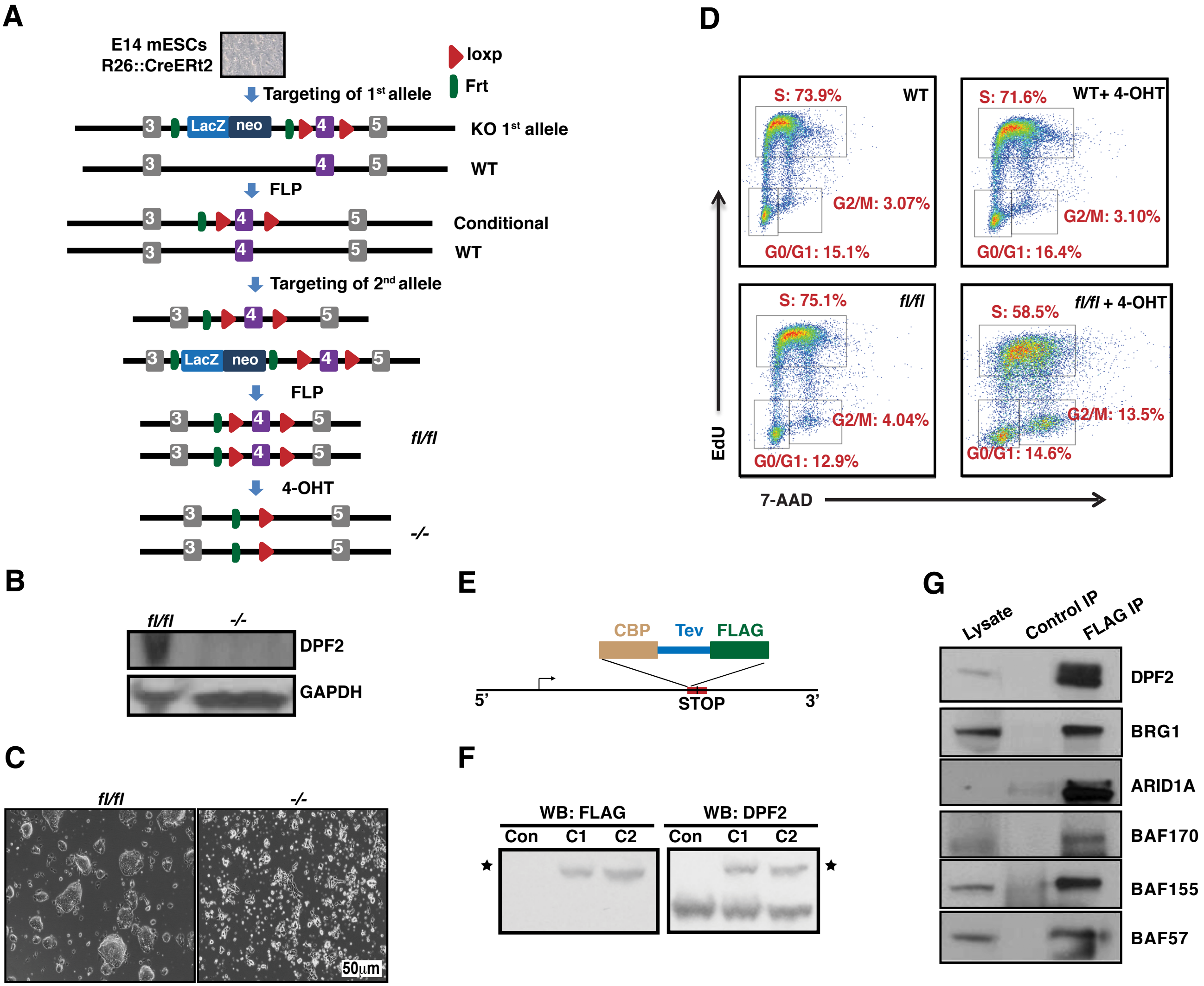

Figure S1. Establishment of *Dpf2* Conditional KO ESCs and Cell Death Induced from *Dpf2* deletion. Related to Figure 1.

(A) Strategy for making *Dpf2* conditional KO ESCs. *Dpf2* targeting vector was first electroporated into R26::CreERT2 E14 ES cells and genotyped by long-range PCR. Next, *Dpf2* heterozygous ES cells were transiently transfected with pCAG-FlpE-IP to delete the LacZ-Neo cassette. The clones with LacZ-Neo cassette popped out were picked up and expanded for a second allele targeting. The second allele was targeted with the same vector for the first targeting. $Dpf2^{fl/fl}$ ES cell lines were made by transiently transfecting with FlpE to pop out the LacZ-Neo cassette.

(B) Deletion of *Dpf2* was induced after treatment with 1μM of 4-OHT for 72 hours and confirmed by Western blot.

(C) Cell death was observed for $Dpf2^{fl/fl}$ and $Dpf2^{-/-}$ cells cultured in N2B27 medium supplemented with BMP4 and Lif medium when treated with 4-OHT for 48h.

(D) Representative EdU/7-AAD cell cycle analysis of $Dpf2^{fl/fl}$ ESCs and WT control cells treated with 4-OHT or ethanol. Relative percentages of phase-specific populations were shown. Data are the typical representative of duplicates.

(E,F) A strategy to fuse the TAP-tag to the C-terminal of *Dpf2* gene (E) and the C-TAP-*Dpf2* clones (C1 and C2) were confirmed by western blot with DPF2 and FLAG antibodies (F). * indicates FLAG-DPF2.

(G) Immunoprecipitation confirmed the interaction of DPF2 with BAF components, BRG1, ARID1A, BAF170, BAF155 and BAF57.

Supplementary Data Figure 2 (related to Figure 2)

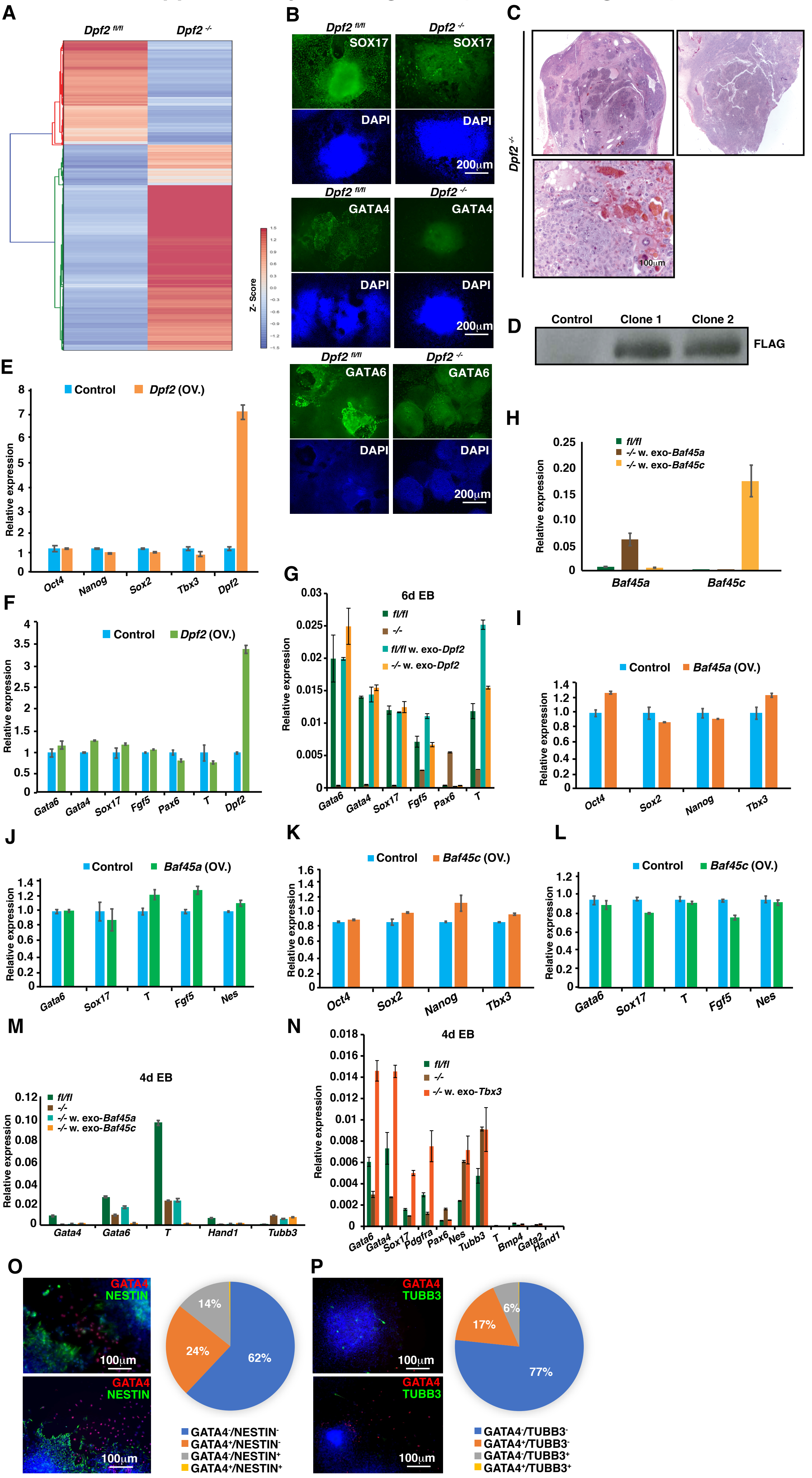

Figure S2. Knockout *Dpf2* impaired the differentiation of ESCs. Related to Figure 2.

(A) Heat map depicts the changes in gene expression between *Dpf2*$^{fl/fl}$ and *Dpf2*$^{-/-}$ ESCs. Heat map showing the log2 values of the relative fluorescence intensity of 1136 genes affected by the loss of *Dpf2*.

(B) Differentiation of *Dpf2*$^{-/-}$ ESCs to primitive endoderm was impaired. The immunostaining of endoderm differentiated from *Dpf2*$^{fl/fl}$ and *Dpf2* $^{-/-}$ ESCs with indicated antibodies. Four day EBs were induced from *Dpf2* $^{fl/fl}$ and *Dpf2* $^{-/-}$ ESCs, and then plated in ES medium without lif in gelatin-coated plates for another 2 days before the immunostaining.

(C) 4-week-old teratoma generated from *Dpf2*$^{-/-}$ ESCs and HE-stained sections showing *in vivo* differentiation defects. Magnification of 16 times (upper two panels); Magnification of 400 times (lower one panel). Upper two panels show predominantly immature neuroepithelium, and bottom panel shows trophectoderm differentiation (trophoblast giant cells, bottom right).

(D) *Dpf2*$^{fl/fl}$ ES cells were transfected with pCAG-flag-*Dpf2*–IZ and selected with zeocin. Single colonies were picked up and confirmed by Western blot with flag antibody.

(E-F) qPCR analysis confirmed that overexpression of *Dpf2* did not affect the expression of pluripotency TFs in ESCs (E) and the expression of lineage markers upon differentiation in EBs for 6 days (F).

(G) Expression of exogenous *Dpf2* restored the differentiation defects of *Dpf2*$^{-/-}$ ESCs. qPCR analysis of the expression of markers in day 6 EBs from *Dpf2* $^{fl/fl}$, *Dpf2*$^{-/-}$, *Dpf2* $^{fl/fl}$ with exo-*Dpf2* and *Dpf2*$^{-/-}$ with exogenously expressed *Dpf2*. Error bars indicate SD of three technical qPCR replicates from a representative experiment.

(H) qPCR analysis confirmed the over-expression of *BAF45a* and *BAF45c* in *Dpf2*$^{-/-}$ ESCs.

(I-L) qPCR analysis confirmed that overexpression of either *Baf45a* or *Baf45c* did not affect the expression of pluripotency of TFs (I and K) and the differentiation of ESCs in day 6 EBs (J and L).

(M) Expression of exogenous *BAF45a* or *BAF45c* did not restored the differentiation defects of *Dpf2*$^{-/-}$ ESCs. qPCR analysis of the expression of marker genes in day 4 EBs from *Dpf2* $^{fl/fl}$, *Dpf2*$^{-/-}$ and *Dpf2*$^{-/-}$ with exogenously expressed *BAF45a* or *BAF45c*.

(N) Expression of exogenous *Tbx3* partially restored the differentiation defects of *Dpf2*$^{-/-}$ ESCs. qPCR analysis of the expression of marker genes in day 4 EBs from *Dpf2*$^{fl/fl}$, *Dpf2*$^{-/-}$ and *Dpf2*$^{-/-}$ with exogenously expressed *Tbx3*.

(O-P) Immunostaining of differentiated *Dpf2*$^{-/-}$ ESCs with exogenous *Tbx3* with indicated antibodies. Nestin positive and Tubb3 positive cells are indicated in Green; Gata4 positive cells are indicated in Red; DAPI (Blue). 4 day EBs were cultured in non-lif ES medium in gelatin-coated plates for 2 days before immunostaining. Different population of cells were quantitated by image analyses. 992 nuclei (O) and 510 nuclei (P) were counted from five and four different fields of view, respectively.

Supplementary Data Figure 3 (Related to Figure 3)

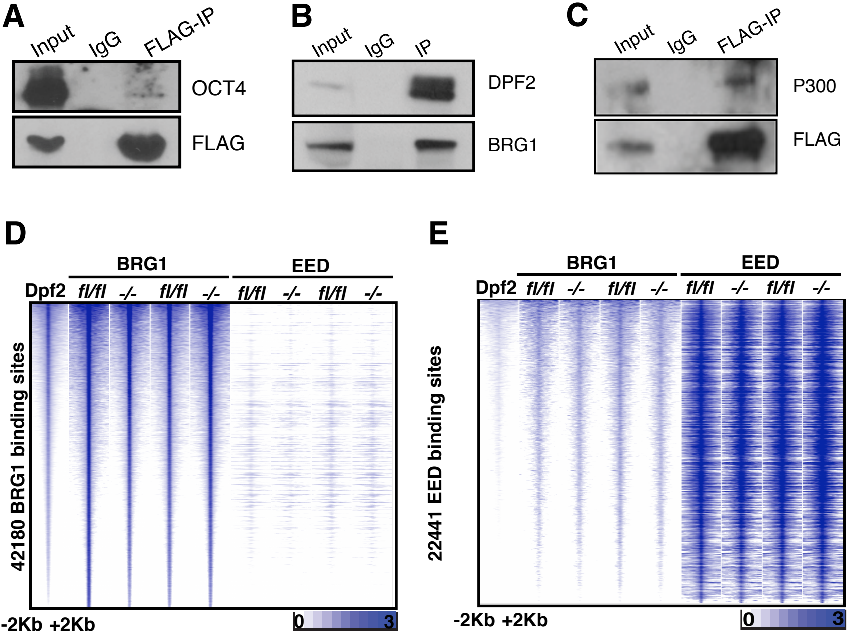

Figure S3. The interactive partners of DPF2 and the effect on BRG1 and EED binding upon the loss of *Dpf2*. Related to Figure 3.

(A) Protein-protein interaction between OCT4 and DPF2.

(B) Protein-protein interaction between BRG1 and DPF2.

(C) Protein-protein interaction between P300 and Flag-DPF2.

(D) Heat map of read normalized tag density profiles at all BRG1 binding sites defined from *Dpf2*$^{fl/fl}$ ESCs. Normalized signal intensities of Dpf2, Brg1 and Eed at 2kb regions flanking the BRG1 binding sites are shown for two independently derived *Dpf2*$^{fl/fl}$ ESC lines and their *Dpf2* knockouts.

(E) As in (D) centering around EED binding sites defined from *Dpf2*$^{fl/fl}$ ESCs.

Supplementary Data Figure 4 (related to Figure 4)

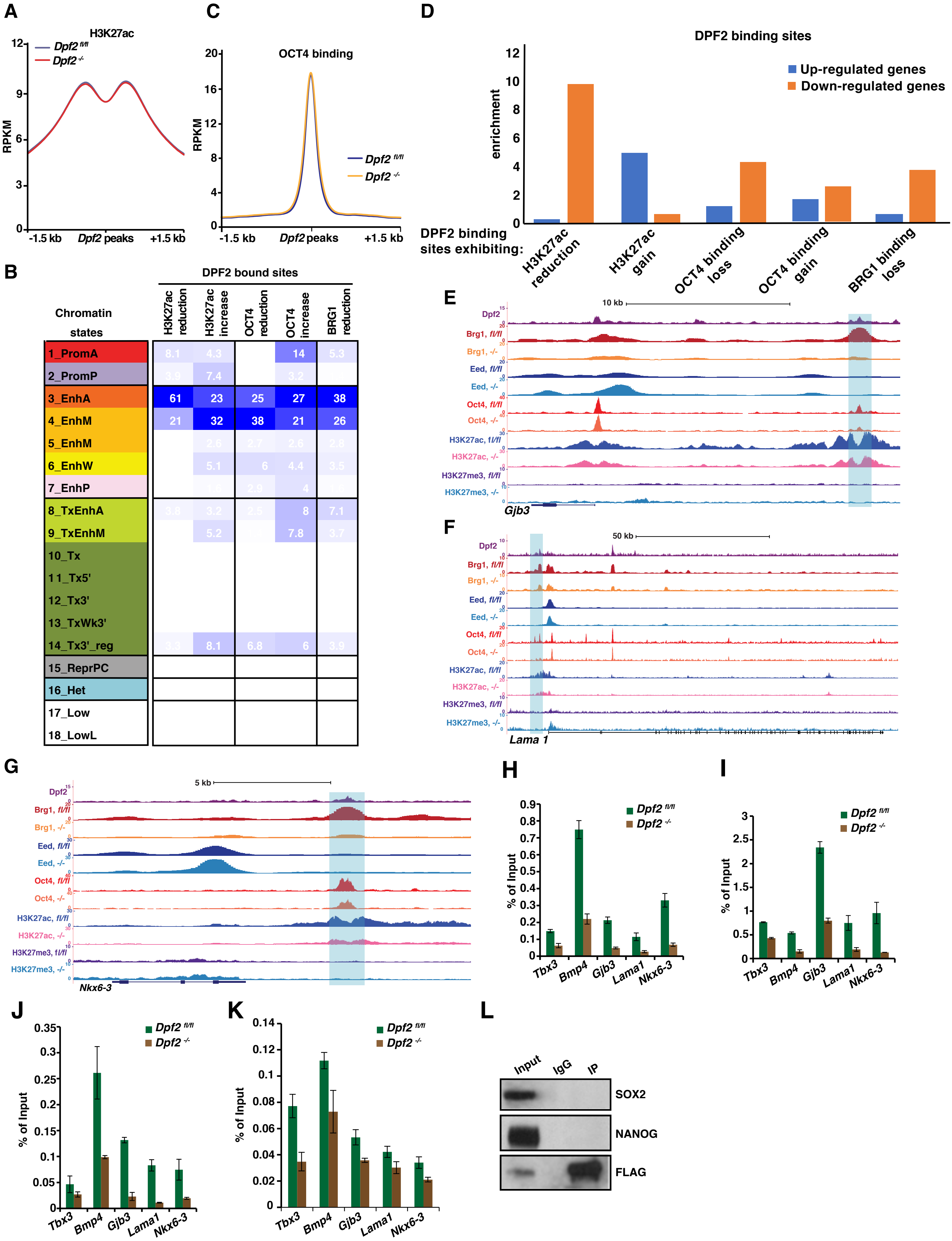

Figure S4. Knockout *Dpf2* alters the H3K27ac modification. Related to Figure 4.

(A) Metaplots of signal intensities for H3K27ac in ESCs at sites bound by DPF2 in ESCs for cells expressing *Dpf2* (blue) or not expressing *Dpf2* (red).

(B) Enrichments of DPF2 bound sites at locations exhibiting loss or gain of H3K27ac, OCT4 or BRG1 binding against annotated chromatin states defined in Figure 3B. Color coding from blue (highest frequency) to white (lowest frequency).

(C) Metaplots of signal intensities for OCT4 in ESCs at sites bound by DPF2 in ESCs for cells expressing *Dpf2* (blue) or not expressing *Dpf2* (yellow).

(D) Enrichment of DPF2 binding sites defined in Figure 4B, 4E and 4I displaying loss or gain of H3K27ac, OCT4 or BRG1 binding in genes down- or up-regulated following *Dpf2* deletion.

(E-G) Genome browser representation of ChIP-seq tracks for Dpf2, Brg1, Eed, Oct4, H3K27me3 and H3K27ac in $Dpf2^{fl/fl}$ and $Dpf2^{-/-}$ ESCs at (E) *Gjb3*; (F) *Lama1*; (G) *Nkx6-3* loci. Highlighted area demarcates an upstream enhancer region with affected BRG1, OCT4 and H3K27ac binding upon loss of *Dpf2*

(H-K) ChIP-qPCR analyses of *Tbx3*, *Bmp4, Gjb3*, *Lama1* and *Nkx6-3* loci from $Dpf2^{fl/fl}$ and $Dpf2^{-/-}$ ESCs were carried out with OCT4 (H), P300 (I), SOX2 (J) and NANOG (K) antibodies. The qPCR amplified regions for *Gjb3*, *Lama1* and *Nkx6-3* were the highlighted region in S4E-S4G. Data are normalized to input values and calculated as percent input recovery. Error bars indicate SD of three technical qPCR replicates from a typical experiment.

(L) Validation of the DPF2-SOX2-NANOG interaction by co-IP.

Supplementary Data Figure 5 (related to Figure 5)

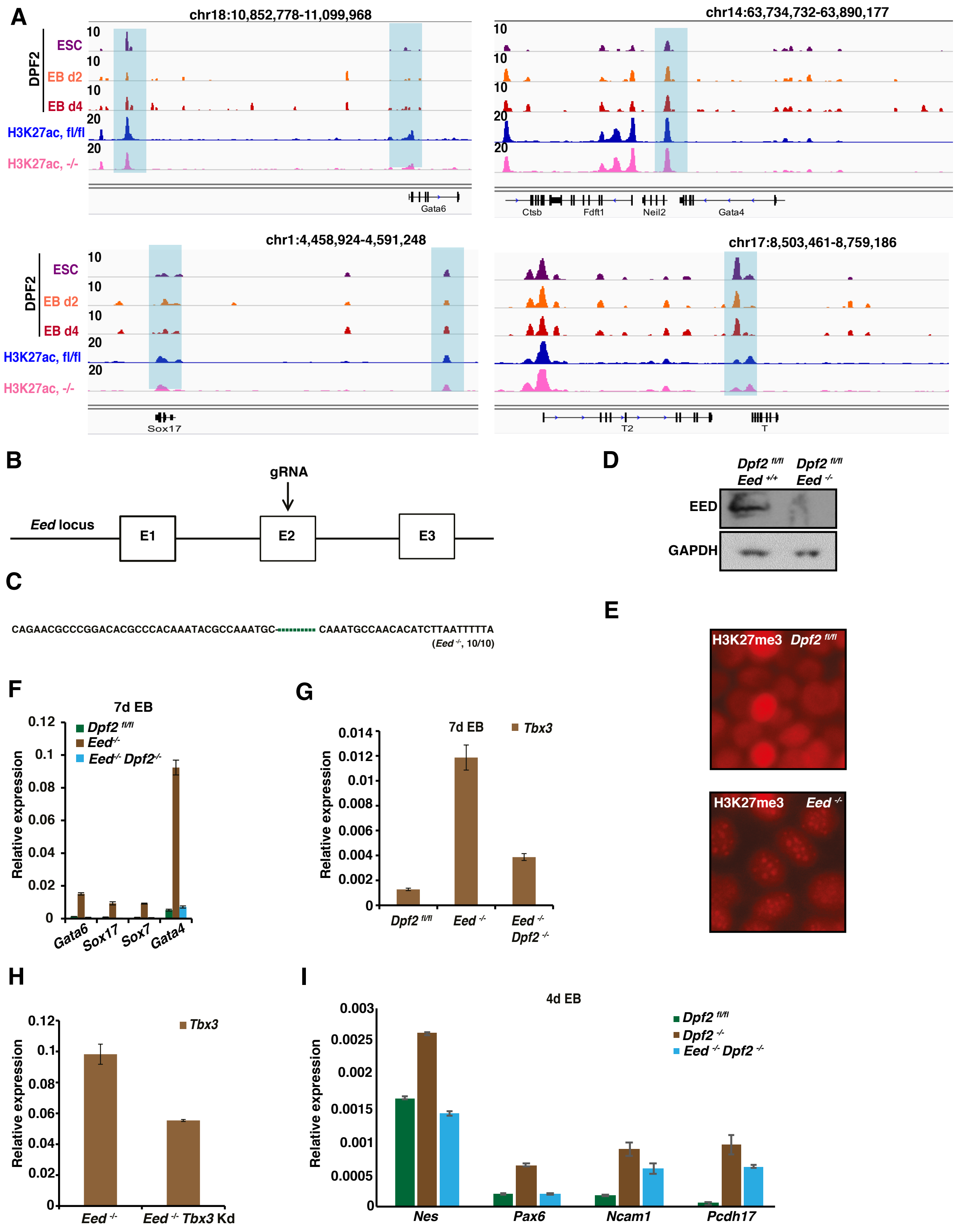

Figure S5. *Dpf2* and *Eed* regulate the endoderm and mesoderm differentiation by regulating *Tbx3* expression. Related to Figure 5.

(A) Endoderm and mesoderm marker gene loci (clockwise, *Gata6*, *Gata4*, *Sox17*, *T*) with the corresponding DPF2 binding events at ESCs and differentiating EBs (day 2 and day4). H3K27ac levels are indicated for wt ESCs (fl/fl) and ESCs with deleted *Dpf2* (KO). Significant DPF2 binding events are indicated in blue boxes.

(B) Strategy for making *Eed* mutant ESCs by CRISPR/Cas9 technology. Two gRNAs were designed and electroporated with Cas9 plasmid to induce the deletion of exon 2 and intron 2 regions.

(C) Confirmation of the deletion of exon 2 and intron 2 regions of *Eed* gene in two of the *Eed* KO clones by Sanger Sequencing. The green dashed lines indicate the deleted regions by CRISPR/Cas9.

(D) Western Blot analysis confirmed the deletion of *Eed* in ESCs.

(E) Immunostaining with anti-H3K27me3 of *Dpf2*$^{fl/fl}$ and *Eed*$^{-/-}$ ESCs. The images were taken under the same exposures.

(F) *Dpf2* and *Eed* opposingly regulate the differentiation of ESCs to endoderm. qPCR analyses of the expression of endoderm marker genes in day 7 EBs from *Dpf2*$^{fl/fl}$, *Eed*$^{-/-}$ and *Eed*$^{-/-}$/*Dpf2*$^{-/-}$ ESCs. Error bars indicate SD of three technical qPCR replicates from a representative experiment.

(G) *Dpf2* and *Eed* opposingly regulate *Tbx3* expression during differentiation. qPCR analyses of *Tbx3* expression in day 7 EBs from *Dpf2*$^{fl/fl}$, *Eed*$^{-/-}$ and *Eed/Dpf2* double KO ESCs.

(H) *Tbx3* expression was knocked down by *Tbx3* shRNA in *Eed*$^{-/-}$ ESCs.

(I) *Dpf2* and *Eed* opposingly regulate the differentiation of ESCs to neuroectoderm. qPCR analyses of the expression of neuroectoderm marker genes in day 4 EBs from *Dpf2*$^{fl/fl}$, *Dpf2*$^{-/-}$ and *Eed/Dpf2* double KO ESCs.

Supplementary Data Figure 6 (relted Figure 5)

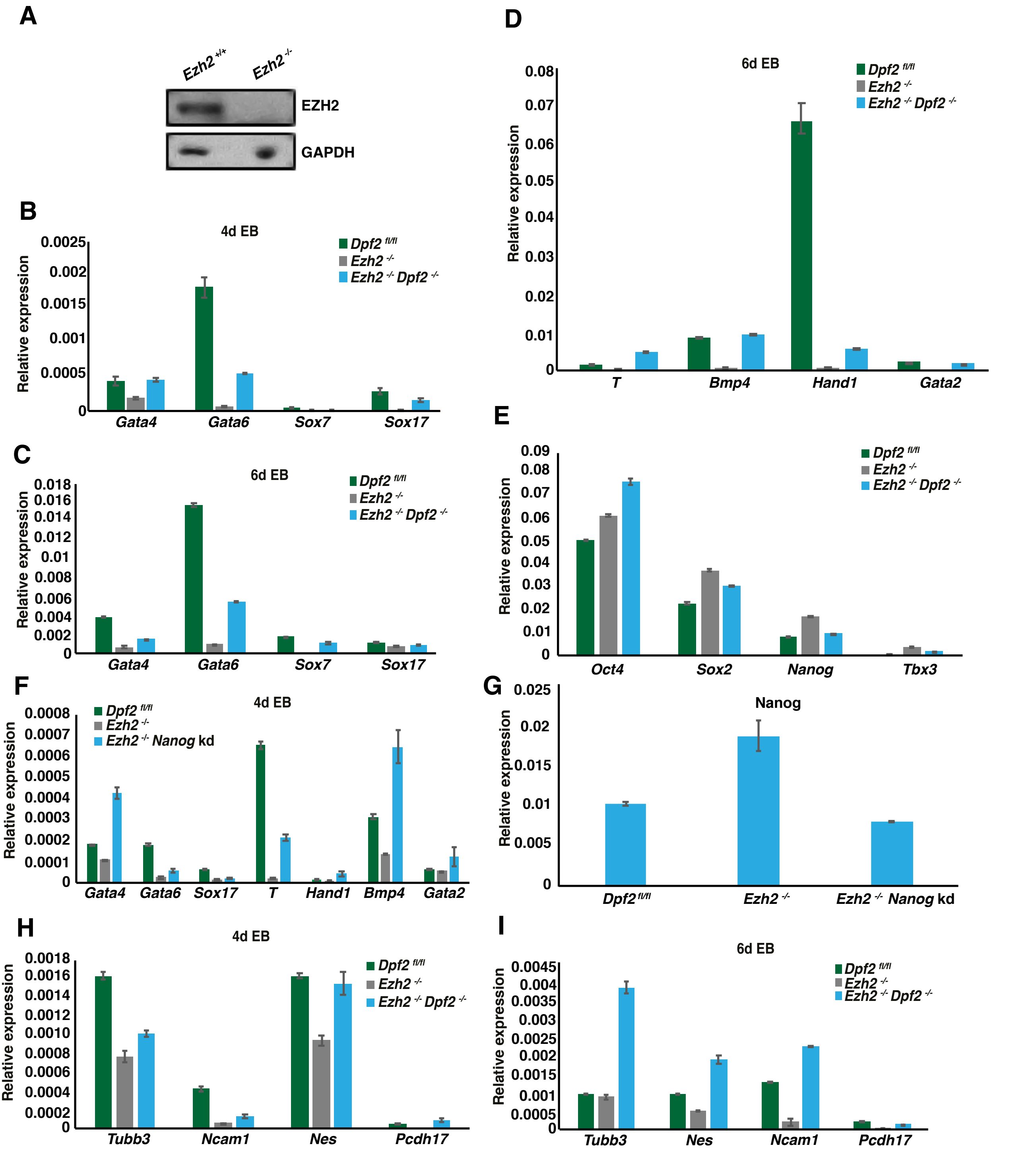

Figure S6. Opposing regulation of ESC differentiation by *Dpf2* and *Ezh2*. Related to Figure 5.

(A) Western Blot analysis confirmed the knockout of *Ezh2* in ESCs.

(B-C) *Dpf2* and *Ezh2* opposingly regulate the differentiation of ESCs to endoderm. qPCR analyses of the expression of endoderm marker genes in day 4 and day 6 EBs from $Dpf2^{fl/fl}$, $Ezh2^{-/-}$ and $Ezh2^{-/-}/Dpf2^{-/-}$ ESCs. Error bars indicate SD of three technical qPCR replicates from a representative experiment.

(D) *Dpf2* and *Ezh2* opposingly regulate the differentiation of ESCs to mesoderm. qPCR analyses of the expression of mesoderm marker genes in day 6 EBs from $Dpf2^{fl/fl}$, $Ezh2^{-/-}$ and $Ezh2^{-/-}/Dpf2^{-/-}$ ESCs.

(E) qPCR analyses of pluripotent associated genes in $Dpf2^{fl/fl}$, $Ezh2^{-/-}$ and *Ezh2/Dpf2* double KO ESCs.

(F) qPCR analyses of the expression of endoderm and mesoderm marker genes in day 4 EBs from $Dpf2^{fl/fl}$, $Ezh2^{-/-}$ and $Ezh2^{-/-}$/*Nanog* kd ESCs.

(G) *Nanog* expression was knocked down by *Nanog* shRNA in $Ezh2^{-/-}$ ESCs.

(H-I) *Dpf2* and *Ezh2* opposingly regulate the differentiation of ESCs to neuroectoderm. qPCR analyses of the expression of neuroectoderm marker genes in day 4 (H) or day 6 (I) EBs from $Dpf2^{fl/fl}$, $Ezh2^{-/-}$ and *Ezh2/Dpf2* double KO ESCs.

Supplementary Data Figure 7 (related to Figure 6)

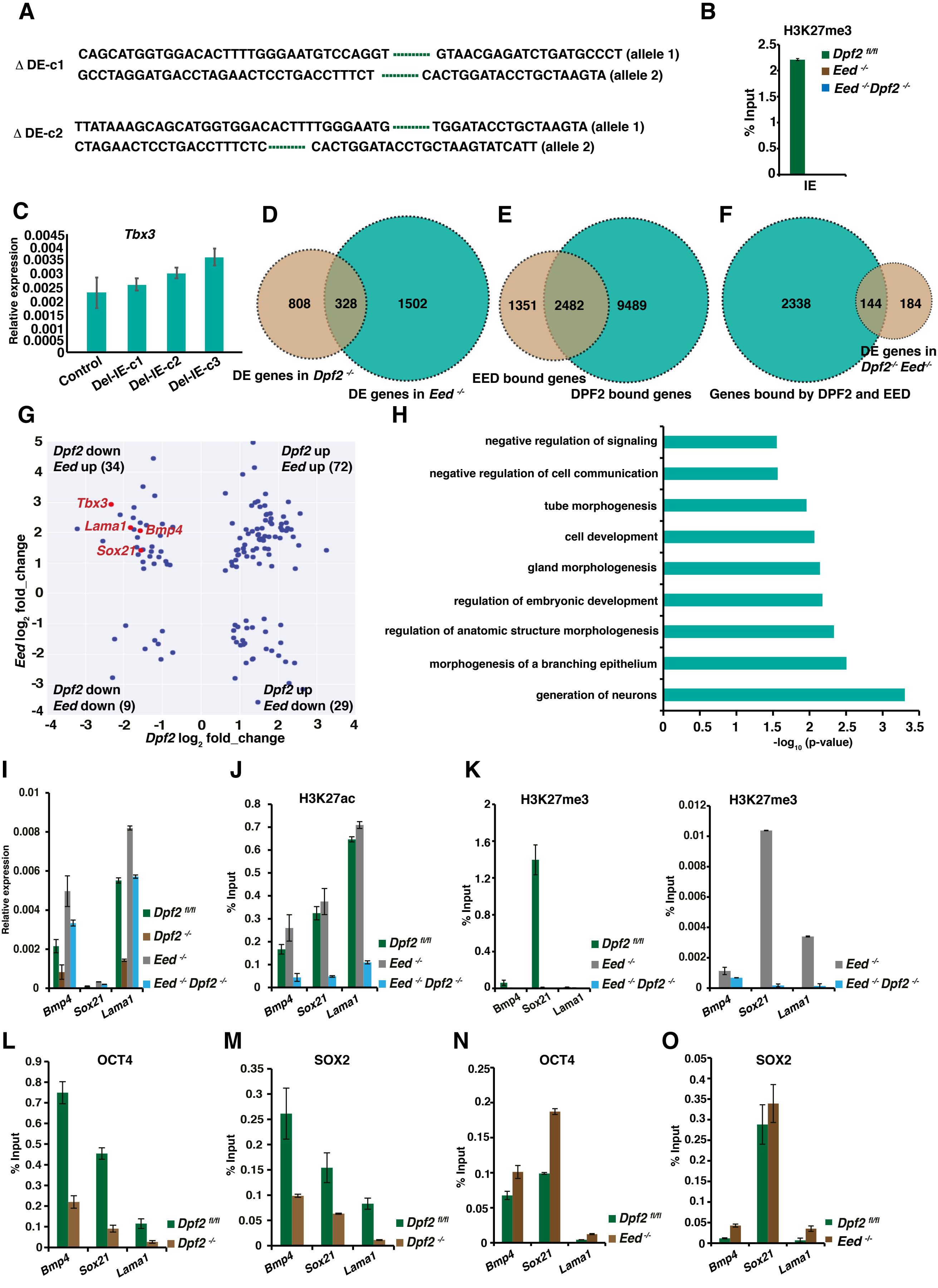

Figure S7. *Dpf2* and *Eed* regulate gene expression oppositely via modulating their H3K27ac and H3k27me3, respectively. Related to Figure 6.

(A) Confirmation of the deletion of DE of *Tbx3* gene by Sanger sequencing. The dashed lines indicate the deleted DE region by CRISPR/Cas9 from two DE KO clones, ΔDE Clone 1 and ΔDE Clone 2.

(B) ChIP-qPCR analyses of the IE loci of *Tbx3* gene from *Dpf2*$^{fl/fl}$, *Eed*$^{-/-}$ and *Dpf2/Eed* double KO ESCs were carried out with H3K27me3 antibody. Data are normalized to input values and calculated as percent input recovery.

(C) qPCR analysis of *Tbx3* expression in *Dpf2* $^{fl/fl}$ and three clones with the IE of *Tbx3* gene deleted.

(D) Venn diagram depicting the number of genes that are differentially expressed in *Eed* and *Dpf2* KO ESCs.

(E) Venn diagram depicting the number of genes that are targeted by *Eed* and *Dpf2* from *Eed* ChIP-seq and Dpf2 ChIP-seq analyses.

(F) Pairwise comparisons of the number of genes misregulated (p_value <0.05) in both *Dpf2*$^{-/-}$ and *Eed*$^{-/-}$ ESCs with which of DPF2 and EED co-bound genes.

(G) The 'four-way' plot for the log2 fold change of the 144 genes (J) in *Eed*$^{-/-}$ and *Dpf2*$^{-/-}$ ESCs. *Tbx3*, *Bmp4*, *Sox21* and *Lama1* are the four of 34 genes opposingly regulated by *Dpf2* and *Eed*.

(H) GO ontology analysis for biological processes associated with genes opposingly regulated by *Dpf2* and *Eed*.

(I) qPCR analyses of the expression of *Bmp4*, *Sox21* and *Lama1* genes in *Dpf2*$^{fl/fl}$, *Dpf2*$^{-/-}$, *Eed* $^{-/-}$ and *Eed*$^{-/-}$ /*Dpf2*$^{-/-}$ ESCs.

(J-K) ChIP-qPCR analyses of the *Bmp4*, *Sox21* and *Lama1* loci from *Dpf2* $^{fl/fl}$, *Eed* $^{-/-}$ and *Eed/Dpf2* double KO ESCs were carried out with H3K27ac (J) and H3K27me3 (K) antibodies. Data are normalized to input values and calculated as percent input recovery.

(L-M) ChIP-qPCR analyses of the *Bmp4*, *Sox21* and *Lama1* loci from *Dpf2* $^{fl/fl}$ and *Dpf2*$^{-/-}$ ESCs were carried out with OCT4 (L) and SOX2 (M) antibodies. Data are normalized to input values and calculated as percent input recovery.

(N-O) ChIP-qPCR analyses of the *Bmp4*, *Sox21* and *Lama1* loci from *WT* and *Eed*$^{-/-}$ ESCs were carried out with OCT4 (N) and SOX2 (O) antibodies. Data are normalized to input values and calculated as percent input recovery. Error bars indicate SD of three technical qPCR replicates from a typical experiment.

Table S3. Related to Figure 2. Down- and Up- regulated genes during EB formation from 4-OHT treated *Dpf2*$^{fl/fl}$ ESCs. Related to Figure 2.

Down-regulated genes during EB formation from 4-OHT treated *Dpf2*$^{fl/fl}$ mES cells			
Down-regulated genes	Neuronal genes	D7	*Viaat, Espn, Relnd*
	Mesoderm	D4	*T, Esam1, Cdx2, Mylpf, Msxl*
		D7	*Hbb-bh1, Apoa2, Esam1, Hand1, Nrp, Efna1, Gata2, Sox18, Hoxd4, Dlk1, Hobx5, Hoxa5, BMP4, Hobx1, Hey2, Foxf1a, Hoxd9, Krt1-18, Foxf1a, Twist2, Tbx1, Isl1, Wt1, Egfl7, Zfpm1, Hoxc6, Mesp1, Krt1-19, Foxc2, Ihh, Krt2-8*
	Endoderm	D4	*Ttr, Pga5, Ctsh, Amn, Ctsc, Apoa1, Hapln1, S100a1*
		D7	*Ttr, Spink3, Amn, Pga5, Sox7, Ctsc, Afp, Slc39a4, Apoa1, H19, Pygl, Apoc1, Rbp4, GATA6, Foxa1, Sox17, Aldh1a2, Cfc1, Ndrg1, Nos3, Pdgfra, FGF10, Cer1, Hhex, Lama1*
	Pluripotent genes	D4	*Sp5, Dnmt3l, Esrrb, Dppa3*
		D7	*Dnmt3l, Tbx3*
Up-regulated genes	Neuronal genes	D4	*Cbln1, Foxd4, Gsh1, Pcdh17, Nkx2, Tcfap2a*
		D7	*Pcdh17, Hes5, Epha7, Catnd2, Epha3, Pou4f1, 2610109H07Rik, Idb4, Gprin1, Pak3, Nefm, Irx2, Rtn1, Scg3, Foxg1, Sytl2, Cav1, Hap1, Edn1, Tcfap2b, Sst, Cplx2, Dcx, Zic4, Bmpr1b, Otx1, Ada, Egr2, Astn1, Kif1b, Mylk, Acta2, Jam2, Rtn1, Zic1, Nes*
	Mesoderm	D4	*Hoxa5, Agpt, Lix1, Dtna*

Table S5. List of qRT-PCR primers and gRNA target sequences used in this study. Related to STAR methods.

**Primers for ChIP-qPCR**		
Gene (ID)	Forward	Reverse
Bmp4	TGGCAAACCGGAAGTTTAAT	CCCTTCCTGCACTTTCAAAT
Nkx6-3	CATGGGTGAGTAGGATTCCC	ACAATAGCTGAGGTGTGGCA
Gjb3	TGAAGGACGATAGCACAAGG	TGGCCACTCCTAGAACATCA
Lama1	CCTCAGCTCCAAGAAAGGAG	GGCATCAGCAGGATGATCTA
Tbx3 (for DE)	CTCAATTCTCTGCCCTTTGG	AAGTCCGGGAAACTCCAATC
Tbx3 (for IE)	CAGGGTTACATAGACAATAA ACCG	ACAGCCTGACTACAGAGTGAAAG T
**CRISPR sgRNA sequences**		
Gene (ID)	gRNA sequence	
Eed	GGTGCATTTGGCGTATTTGTGG (sgRNA1)	
Eed	AAGCTGGGGAAAGGGAAAATGG (sgRNA2)	
Tbx3 (DE)	TCTTGAAGGTTACATATACATGG (sgRNA1)	
Tbx3 (DE)	ATGTAGATAAACCCAGAGACTGG (sgRNA2)	
Tbx3 (DE)	GCATCAGATCTCGTTACGGATGG (sgRNA3)	
Tbx3 (DE)	GAGGGCATCAGATCTCGTTACGG (sgRNA4)	
Tbx3 (IE)	TTCCTCGGAAGTTTCGTGGTGGG (sgRNA1)	
Tbx3 (IE)	TCTGTTCCTCGGAAGTTTCGTGG (sgRNA2)	
Tbx3 (IE)	GTAAAATGGGACGATGATAATGG (sgRNA3)	
Tbx3 (IE)	ATACACTTTACACTATCAGAAGG (sgRNA4)	
Ezh2	CACATTTAAGTCTCACACCGAGG (sgRNA1)	
Ezh2	ACATTTAAGTCTCACACCGAGGG (sgRNA2)	
Ezh2	TTAGTCCAGCCGGGCGGTGTTGG (sgRNA3)	
Ezh2	GTTTATTTAGTCCAGCCGGGCGG (sgRNA4)	
**Primers for qPCR**		
Baf45a forward primer	GGCTCAGGAGACAGCTCAAG	
Baf45a reverse primer	TTCCGCTTGAAGGAGGTCAC	
Baf 45c forward primer	AAGACCAGGAGACCCGATCC	
Baf45c reverse primer	CAGGCAAGTTGGATGACCAGA	
